# Postmagmatic Tectonic Evolution of the Outer Izu‐Bonin Forearc Revealed by Sediment Basin Structure and Vein Microstructure Analysis: Implications for a 15 Ma Hiatus Between Pacific Plate Subduction Initiation and Forearc Extension

**DOI:** 10.1029/2019GC008329

**Published:** 2019-12-06

**Authors:** W. Kurz, P. Micheuz, G. L. Christeson, M. Reagan, J. W. Shervais, S. Kutterolf, A. Robertson, K. Krenn, K. Michibayashi, D. Quandt

**Affiliations:** ^1^ NAWI Graz Geocenter, Institute of Earth Sciences University of Graz Austria; ^2^ Jackson School of Geosciences University of Texas Institute for Geophysics Austin TX USA; ^3^ Department of Earth and Environmental Science University of Iowa Iowa City IA USA; ^4^ Department of Geological Sciences Utah State University Logan UT USA; ^5^ Dynamics of the Ocean Floor GEOMAR Helmholtz Centre for Ocean Research Kiel Kiel Germany; ^6^ School of Geosciences The University of Edinburgh Edinburgh UK; ^7^ Department of Earth and Planetary Sciences, Graduate School of Environmental Studies Nagoya University Nagoya Japan

**Keywords:** International Ocean Discovery Program (IODP), Expedition 352, Izu ‐ Bonin forearc, faulting and extension, syntectonic sedimentation, hiatus

## Abstract

International Ocean Discovery Program Expedition 352 recovered sedimentary‐volcaniclastic successions and extensional structures (faults and extensional veins) that allow the reconstruction of the Izu‐Bonin forearc tectonic evolution using a combination of shipboard core data, seismic reflection images, and calcite vein microstructure analysis. The oldest recorded biostratigraphic ages within fault‐bounded sedimentary basins (Late Eocene to Early Oligocene) imply a ~15 Ma hiatus between the formation of the igneous basement (52 to 50 Ma) and the onset of sedimentation. At the upslope sites (U1439 and U1442) extension led to the formation of asymmetric basins reflecting regional stretch of ~16–19% at strain rates of ~1.58 × 10^−16^ to 4.62 × 10^−16^ s^−1^. Downslope Site U1440 (closer to the trench) is characterized by a symmetric graben bounded by conjugate normal faults reflecting regional stretch of ~55% at strain rates of 4.40 × 10^−16^ to 1.43 × 10^−15^ s^−1^. Mean differential stresses are in the range of ~70–90 MPa. We infer that upper plate extension was triggered by incipient Pacific Plate rollback ~15 Ma after subduction initiation. Extension was accommodated by normal faulting with syntectonic sedimentation during Late Eocene to Early Oligocene times. Backarc extension was assisted by magmatism with related Shikoku and Parece‐Vela Basin spreading at ~25 Ma, so that parts of the arc and rear arc, and the West Philippine backarc Basin were dismembered from the forearc. This was followed by slow‐rift to postrift sedimentation during the transition from forearc to arc rifting to spreading within the Shikoku‐Parece‐Vela Basin system.

## Key Points

- IODP Expedition 352 obtained a 15 Ma hiatus between igneous basement formation and onset of sedimentation along the Izu ‐ Bonin forearc
- Incipient Pacific Plate rollback is inferred to have triggered forearc extension and syntectonic sedimentation
- Downslope increase of stretch is inferred to be related to upper plate flexure toward the retreating trench

## 1 Introduction

**Figure 1:**
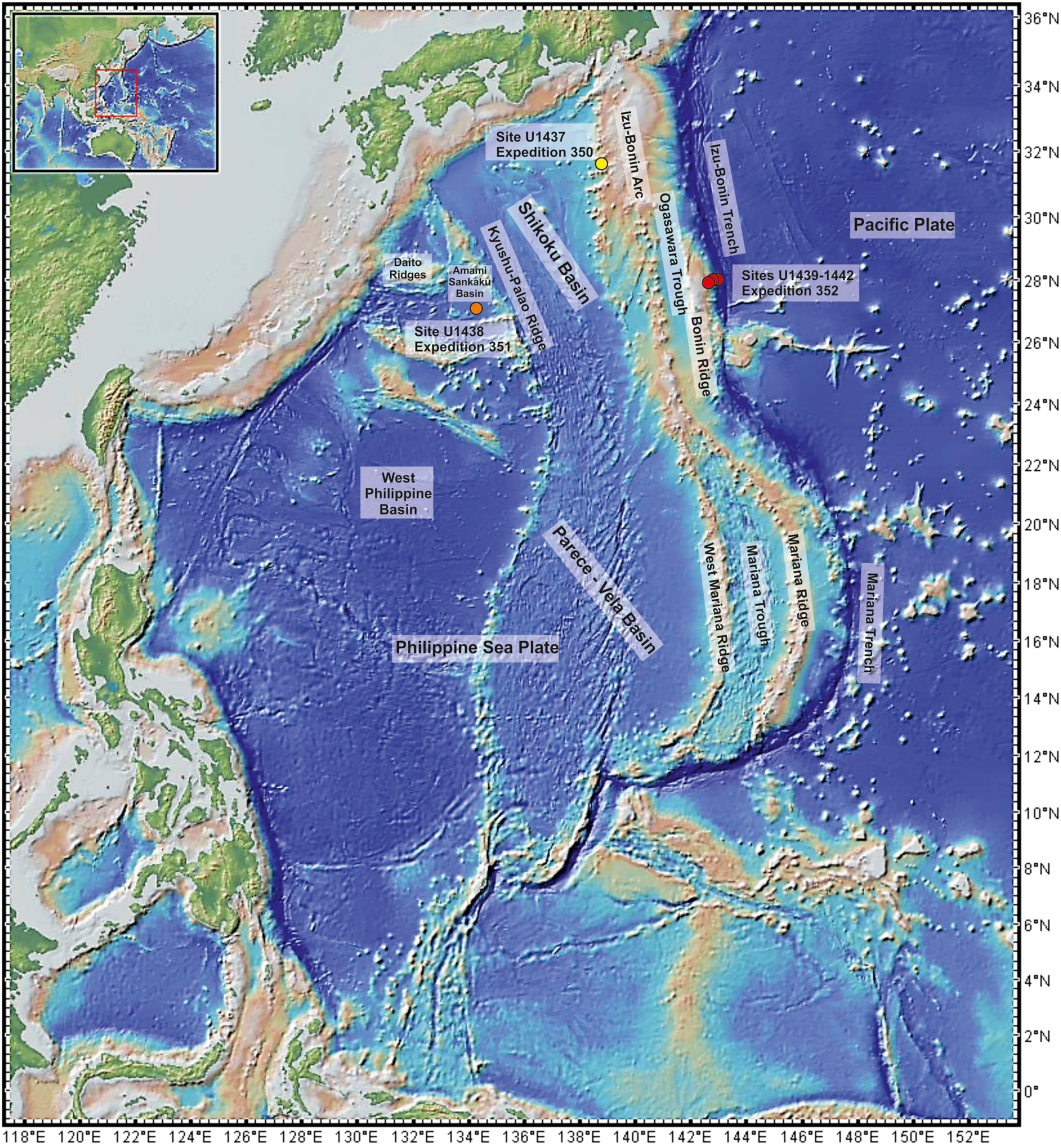
Location map for IODP Expeditions 352, 351, and 350, showing the Izu‐Bonin‐Mariana arc system along the western Pacific margin and the Philippine Sea Plate backarc basins. Red dots show location of the Expedition 352 drill sites; orange and yellow dot shows the drill site for Sister Expeditions 351 and 350, respectively.

In 2014, the International Ocean Discovery Program (IODP) conducted three closely related drilling expeditions using the R/V JOIDES Resolution to explore the Izu‐Bonin‐Mariana (IBM) arc system (Figure 1), and the magmatic processes related to subduction initiation. Expedition 350 (Sites U1436 and U1437) was the first expedition designed to ascertain “the missing half” of the subduction factory in the IBM rear arc (Busby et al., 2017; Tamura et al., 2015). Expedition 351 (Site U1438) drilled west of the Kyushu‐Palau remnant arc ridge with focus on IBM arc origins (Arculus, Ishizuka, Bogus, Gurnis, et al., 2015). Expedition 352 (Sites U1439 to U1442) drilled the igneous outer Bonin forearc, related to subduction initiation (Reagan et al., 2015). The IBM system is the type locality to examine the accretion of oceanic crust immediately after the initiation of subduction, arc evolution, and continental crust formation in a suprasubduction zone (SSZ) setting (Rudnick, 1995; Stern et al., 2003; Stern & Bloomer, 1992; Tatsumi & Stern, 2006). This predominantly submarine convergent plate boundary extends for 2,800 km from the Izu Peninsula to Guam. It resulted from ~52 Ma of subduction of the Pacific Plate beneath the eastern margin of the Philippine Sea plate (Reagan et al., 2019). IODP expedition results were published recently, notably concerning the magmatic evolution (Brandl et al., 2017; Yogodzinski et al., 2018; Hickey‐Vargas et al., 2018; Shervais et al., 2019), and the age of IBM volcanic rocks (Barth et al., 2017; Ishizuka et al., 2018; Reagan et al., 2019). In addition, the drilled cores provided (hemi)pelagic sedimentary‐volcaniclastic successions and tectonic structures that bear information on the tectonic evolution of the outer IBM forearc. The biostratigraphic record within the related sedimentary basins provides temporal constraints on the fault activity, and revealed a ~15 Ma hiatus between the formation of the igneous IBM basement and the onset of sedimentation within these basins.

In this study we investigate the overall structure of fault‐bounded sedimentary basins and the related tectonic structures at IODP Expedition 352 drill sites in order to reconstruct the structural and tectonic evolution of the IBM forearc, in particular the ~15 Ma time gap between Pacific Plate subduction initiation and forearc extension with related sedimentary basin formation. The results from this study also have implications for the evolution of the IBM system at a lithospheric scale and are implemented into an overall tectonic model. The new results from structural and tectonic studies, together with its magmatic and sedimentary inventory, facilitate a comprehensive reconstruction of forearc architecture in a SSZ tectonic setting and are therefore relevant to the interpretation of SSZ ophiolites worldwide.

**Figure 2:**
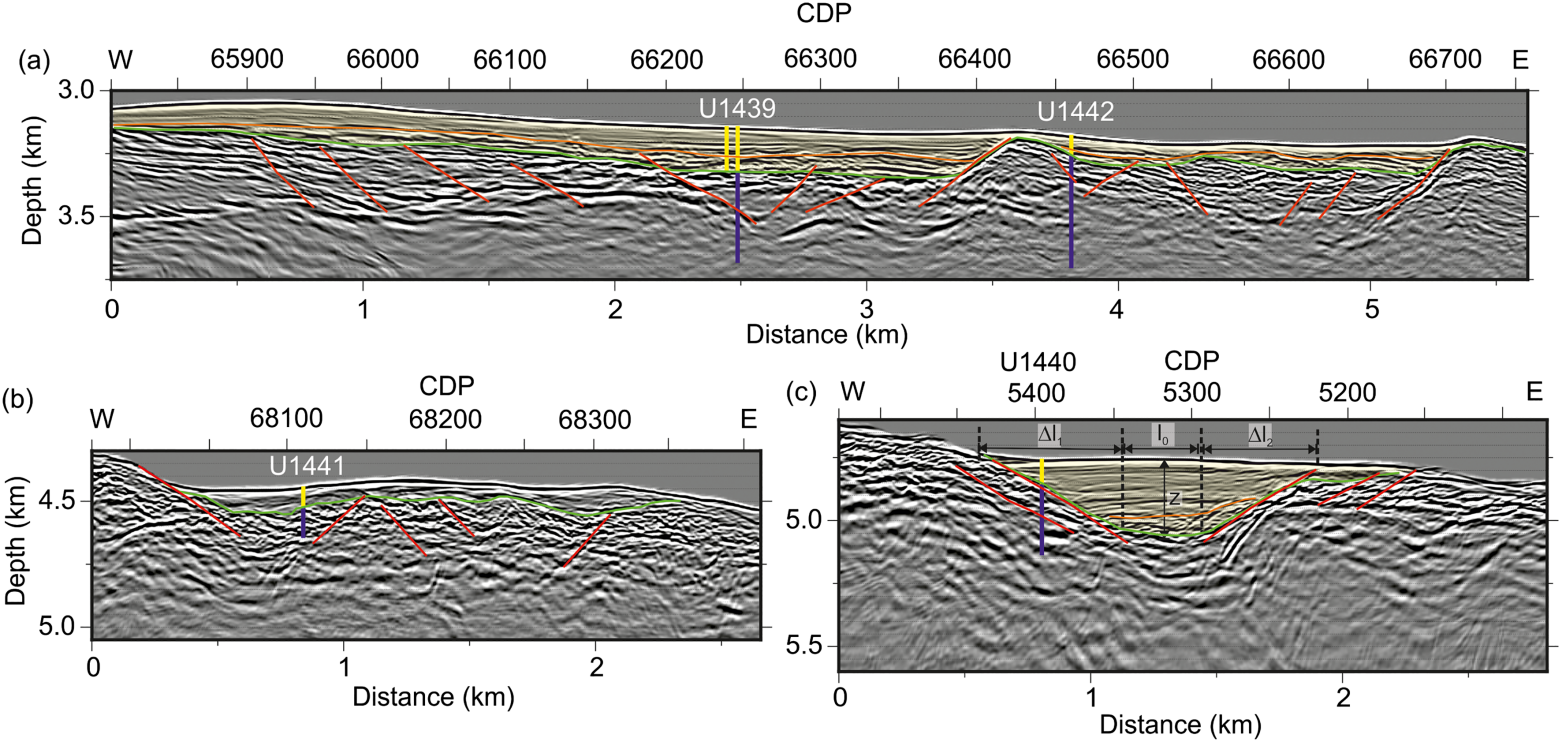
Prestack time migrated images, converted to depth, showing the location of drill sites (after Christeson et al., 2016). (a) Upper forearc basin Sites U1439 and U1442. (b) Lower forearc basin site U1441. (c) Lower forearc basin site U1440. Images are plotted with a 0.5 s automatic gain control, without exaggeration. Sedimentary and basement units at the drill sites are indicated by green and orange lines, respectively; the sedimentary cover is displayed in pale yellow. Interpretation: green lines represent the sediment‐basement interface, the orange lines the change in dip (unconformities) within sedimentary sections, and red lines = normal faults. CDP: Common depth point.

**Figure 3:**
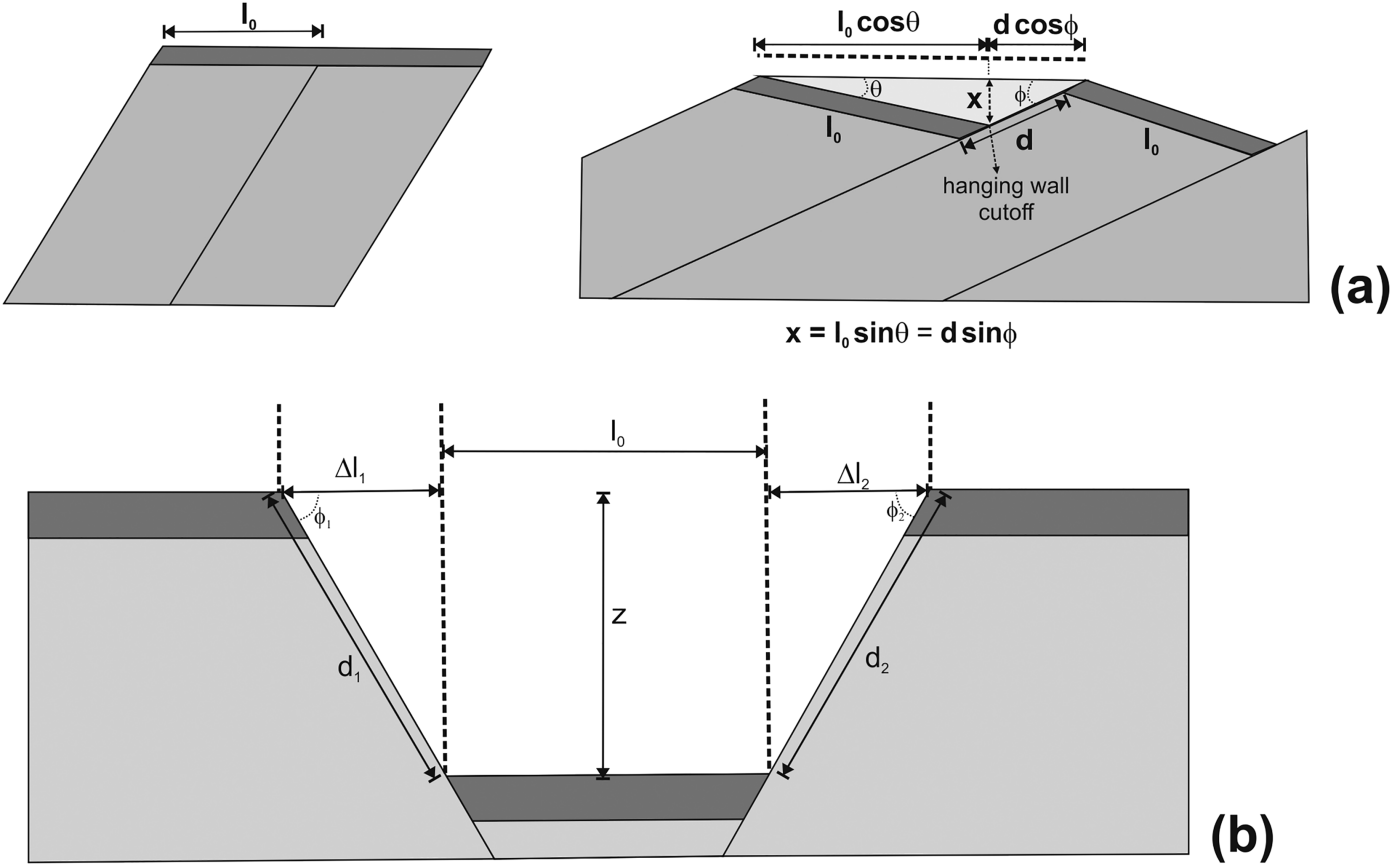
(after Twiss & Moores, 2007): (a) Geometric relationships of equally spaced planar, rotating high‐angle normal faults above a (hypothetical) detachment; ϕ: fault dip angle, θ: sediment dip angle, d: displacement, *l*_0_: initial bed length; and *x*: maximum basin depth (sedimentary cover thickness). (b) Geometric relationship of conjugate, nonrotating high‐angle normal faults; ϕ: fault dip angle, *d*: displacement, *l*_0_: initial bed length, Δ*l*_1,2_: fault‐related extension.

Tectonic structures mainly resulted from brittle deformation that took place within and in the vicinity of fault zones. Timing of fault activity is basically derived from the stratigraphy within the adjacent sedimentary basins, described in detail by Robertson et al. (2018). These fault zones display a wide variety of related deformation structures including discrete faults, cataclastic shear zones, extensional fractures, and veins in which minerals, mainly derived from hydrothermal fluids, precipitated (e.g., Kurz et al., 2015; Reagan et al., 2015). The faults and related veins were created after the formation of the forearc crust. Extension veins also help to elucidate the deformation history of their host rocks and the associated fluid effects (e.g., Bons et al., 2012; Hilgers & Urai, 2002; Ramsay & Huber, 1983). We therefore analyzed calcite vein microstructures from drill cores at the Expedition 352 Sites U1439, U1440, U1441, and U1442. Microstructures obtained from these veins reveal the related deformation conditions, in particular differential stresses, deformation temperatures, and constraints on strain rates. Similar structures were also studied within the magmatic basement of the Amami‐Sankaku Basin (ASB) in the northwest Philippine Sea, drilled during Expedition 351 at Site U1438. The techniques used were electron backscatter diffraction (EBSD) combining with stress piezometry of mechanically formed e‐twins of calcite.

## 2 Geological Background

The approximately north‐south trending IBM arc is related to the west dipping subduction of the Pacific Plate under the eastern margin of the Philippine Sea Plate (PSP) (Figure 1). From north to south the subducting Pacific Plate steepens from about 30° beneath Japan to nearly vertical below the Mariana arc (Faccenna et al., 2018; Holt et al., 2018, and references therein). It is assumed that the IBM subduction zone began as part of a hemispheric‐scale tectonic structure (e.g., a transform fault) of ancient, dense lithosphere in the western Pacific Ocean (Bloomer et al., 1995; Hall et al., 2003; Stern, 2004).

### 2.1 Igneous Basement

The timing of large‐scale lithospheric subduction is constrained by the age of igneous rocks of the IBM forearc that started in the Eocene, at ~52 Ma (Bloomer et al., 1995; Cosca et al., 1998; Ishizuka et al., 2006; Ishizuka et al., 2018; Reagan et al., 2019). The initial spreading generated forearc basalt (FAB) lavas at ~52 Ma; later volcanism produced low‐Si, then high‐Si boninites (~51–46 Ma: Ishizuka et al., 2006, Ishizuka, Tani, et al., 2011; Reagan et al., 2013, Reagan et al., 2019) to form the proto‐Bonin Ridge. Magmatic activity appears to have migrated inboard, with the FAB erupting closest to the trench (Sites U1440 and U1441) and the boninites (Sites U1439 and U1442 and the Bonin Ridge) erupting farther from the trench. Arc andesites erupted in the western part of the Bonin Ridge (Figure 1) after about 46 Ma (Ishizuka et al., 2006). Ishizuka, Taylor, et al. (2011) argue that the crustal stratigraphy of the IBM forearc is oceanic crust overlain by boninitic and later arc lavas, while Stern and Bloomer (1992) and Ishizuka et al. (2006) argue that a broad swath of forearc crust formed by seafloor spreading after subduction initiation. Relating to IODP Expedition 352 results, Reagan et al. (2017, 2019) have developed a hybrid of these models, in which FAB and low‐Si boninite magmas were generated during an initial period of seafloor spreading, whereas the high‐Si boninites erupted subsequently at discrete volcanic centers (i.e., the nascent arc) at 51.3 Ma. The rear arc basalts of the ASB (Site 351‐U1438) are younger than the Sites U1439 and U1442 boninite, which suggests that they formed by renewed volcanism west of the Bonin Ridge, after initial spreading ceased in the forearc (Ishizuka et al., 2018).

The Philippine Sea Plate (PSP) (Figure 1) is characterized by a complex tectonic and magmatic evolution (Hall et al., 1995). It is surrounded by transform faults and subduction zones. Initial seafloor spreading began around the time of subduction initiation at ~52 Ma to form the initial back arc basin in the PSP (i.e., the West Philippine Basin) (Deschamps & Lallemand, 2002; Ishizuka et al., 2018; Seton et al., 2012; Whittaker et al., 2007; Wu et al., 2016). The ASB, in which Site U1438 was drilled, is situated north of the West Philippine Basin and west of the Kyushu‐Palau Ridge. The ASB sole comprises about 1.5 km of sediment overlying igneous oceanic crust. The ASB basement commonly encompasses basaltic sheet flows and dikes of high‐Mg, low‐Ti, tholeiitic basalts showing variable alteration and veining (Arculus, Ishizuka, Bogus, Gurnis, et al., 2015; Arculus, Ishizuka, Bogus, & the Expedition 351 Scientists, 2015). The geochemistry of the basement lavas (Hickey‐Vargas et al., 2018; Yogodzinski et al., 2018) indicates derivation from mantle source rocks that were more melt depleted than those of typical mid‐ocean ridges, similar to the IBM FABs (Shervais et al., 2019). The basaltic basement of the ASB (IODP Site U1438) is currently interpreted as part of the initial basement produced during subduction initiation (Arculus, Ishizuka, Bogus, & the Expedition 351 Scientists, 2015), although ages of the Amami‐Sankaku basement are concurrent with high‐Si boninite volcanism on the Bonin Ridge (Ishizuka et al., 2018), allowing an alternative explanation that this was the first IBM back arc basin (Reagan et al., 2019).

### 2.2 Postmagmatic Faulting and Sedimentation

The arc and forearc crust that formed after subduction initiation was affected by later fault‐related deformation and chemical/hydrothermal alteration owing to tectonic deformation at the IBM forearc. Extension‐related asymmetric sedimentary basins (e.g., half‐grabens) are developed at Sites U1439 and U1442 on the upper trench slope (Christeson et al., 2016; Kurz et al., 2015; Robertson et al., 2018) (Figure 2). The basins are bounded by west dipping normal faults along their eastern margins, accompanied by syntectonic (hemi)pelagic and volcaniclastic sedimentation. According to shipboard data, the lowermost sedimentary units at Sites U1439 and U1442 were tilted eastward by ~20°, and the tilted beds were covered by subhorizontal beds (Reagan et al., 2015). Drill cores revealed discrete shear structures with dominant reverse to oblique reverse slip along subhorizontal fault zones. These were either reactivated as, or transected by, normal faults, oblique faults with a normal slip component, and strike‐slip faults (Reagan et al., 2015). At Sites U1440 and U1441, on the outer forearc, the sedimentary basins are bounded by normal and oblique‐slip/strike‐slip faults. The sedimentary fill was not significantly affected by tectonic tilting. Additional extensional, mainly fault‐related structures are steeply dipping to subvertical mineralized veins and extension fractures.

Biostratigraphic constraints from calcareous nannofossils reveal initial sedimentation at ~35 Ma (Robertson et al., 2018). Tephra layers higher in deep‐sea successions indicate that explosive dacitic IBM volcanism started around 28.6 Ma (Kutterolf et al., 2018). Since the IBM igneous basement formed at 52–50 Ma (Reagan et al., 2019), a ~15 Ma depositional hiatus must therefore exist prior to the oldest known sediment deposits.

The sedimentary‐volcanogenic evolution of the forearc basins, together with their tectonic and paleogeographic implications for the IBM forearc, had recently been discussed by Robertson et al. (2018) and by Kutterolf et al. (2018). Site U1439 has by far the best overall recovery of the sedimentary succession, with approximately 180 m of mainly pelagic sediments and tephras. This site can therefore be taken as reference site for the general upslope structure and related postmagmatic tectonics. Based on biostratigraphic constraints, three major time slices of fine‐grained background sedimentation were defined by Robertson et al. (2018). Time Slice 1 ranges from the early Oligocene (ca. 34.44–32.92 Ma) to early Miocene (ca. 23 Ma), with sedimentation of nannofossil chalk, marl, or limestone, variably mixed with volcaniclastic and tuffaceous sediment (i.e., calcareous siltstone or sandstone) as well as IBM tephras since 16 Ma. The boundary between Time Slices 1 and 2 coincides with the change in sediment dip, from subhorizontal (Time Slice 2) to continuously increasing dip angles below 127 m below sea floor (bsf) (Time Slice 1). This boundary also marks the demise of the early IBM‐derived volcanism. Time Slice 2 ranges from the early Miocene to the mid‐Pliocene (ca. 23–4 Ma), with clay/claystone and mud/mudstone (variably mixed with silt/siltstone and sand/sandstone). Time Slice 3 ranges from mid‐Pliocene to Holocene (ca. 4–0 Ma) with sedimentation of nannofossil ooze and minor mud (variably mixed with tuffaceous sediment and tephras from IBM and mainland Japan).

The succession at Site U1442A has many similarities to that at Site U1439, although thinner, and the Miocene succession is not as complete; no early Miocene nannofossils were identified (Robertson et al., 2018).

For Site U1440, the basal sediments are Oligocene (32.92 Ma), that is, coeval with the basal sediments at Sites U1439 and U1442. A major hiatus ranges from the earliest Oligocene to the Miocene‐Pliocene boundary. Age profiles differ considerably at Site U1441 with late Miocene basal sediments directly overlying the basement. Neither Oligocene nor early Miocene sediments were recovered. Middle Miocene sediments are also missing (Robertson et al., 2018).

## 3 Samples and Methods

During IODP Expedition 352 samples were taken subsequently to shipboard drill core description. The sampling mainly focused on deformational structures (veins and fault rocks) from several drill sites (U1439, U1440, U1441, and U1442) (Figures 1 and 2). The supporting site survey data for Expedition 352 are archived at the IODP Site Survey Data Bank (http://web.iodp.tamu.edu/UWQ/).

Samples were primarily taken from core intervals, which showed both (1) obvious shear deformation along faults zones (cemented cataclasites and fault breccias) and (2) zones characterized by obvious fluid activity in terms of wall‐rock alteration and precipitation within extensional veins, gashes, and voids along shear fractures. Additional samples from IODP Expedition 351 were obtained from the Kochi Core Center (Japan).

Representative samples were cut from the working half of the drill cores into slices, with a maximum size of 10 × 5 cm. Deformation structures were cut parallel to the drill‐core axis (being defined as *Z* axis). Polished thin sections, with a size of ~27 × 46 mm, were used for optical microscopy. About 200 thin sections were studied.

The locations of samples described are listed in Table 1.

**Table 1:** Representative Sample Locations and Sites

Sample	IODP sample ID	Depth below seafloor [m]	Intersecting faults^a^	Vein type	Analyses
BON‐1	352‐U1439C‐13R‐1‐W 42/47	280.5	yes	blocky	piezometer
BON‐2	352‐U1439C‐23R‐1‐W 109/113	359.2	yes	blocky	piezometer
BON‐3	352‐U1439C‐23R‐2‐W 15/21	359.4	yes	blocky	piezometer
BON‐4	352‐U1439C‐26R‐2‐W 9/11	388.9	yes	blocky	piezometer
BON‐5	352‐U1439C‐27R‐1‐A 100/118	398	yes	—	microstructure
BON‐6	352‐U1439C‐27R‐4‐W 25/30	401	yes	blocky	piezometer
BON‐7	352‐U1439C‐29R‐4‐W 60/63	421.1	yes	blocky	piezometer
BON‐8	352‐U1439C‐31R‐3‐W 66/69	439.4	yes	blocky	piezometer
BON‐9	352‐U1439C‐32R‐3‐W 113/119	449.5	no	blocky	EBSD
BON‐10	352‐U1439C‐32R‐4‐W 111/114	451	no	blocky	piezometer
BON‐11	352‐U1439C‐33R‐2‐W 31/34	457.3	no	blocky	piezometer
BON‐12	352‐U1439C‐43R‐1‐A 25/41	524.1	yes	—	microstructure
FAB‐1	352‐U1440B‐12R‐1‐W 145/149	165.1	no	blocky	piezometer
FAB‐2	352‐U1440B‐17R‐1‐W 58/63	212.8	no	blocky	piezometer
FAB‐3	352‐U1441A‐14R‐1‐W 129/131	123.1	no	blocky	EBSD
FAB‐4	352‐U1441A‐20R‐1‐W 22/24	180.4	yes	—	microstructure
ASB‐1	351‐U1438E‐66R‐2‐W 26/30	1448.2	—	blocky	piezometer
ASB‐2	351‐U1438E‐71R‐3‐W 67/74	1473.4	—	blocky	piezometer
ASB‐3	351‐U1438E‐82R‐2‐W 43/52	1553.8	—	blocky	piezometer/EBSD

a From IODP shipboard observations.

### 3.1 Seismic Images

Seismic images across Expedition 352 sites from Christeson et al. (2016), complimented with interpreted faults, unconformities, and sedimentary bedding are displayed in Figure 2. The data are prestack migrated images, converted to depth using velocities of 1,500 m/s for the water column, 1,700 m/s for sediments, and the appropriate crustal velocities from a coincident seismic refraction profile (Christeson et al., 2016). The sediment‐basement interface is indicated by a change from continuous reflectors to lower‐frequency, disrupted reflectors, as confirmed, where possible, by the depth of the drilled igneous basement‐sediment contact. Within the sedimentary succession we obtain a change from shallow, subhorizontal reflectors to more chaotic, dipping reflectors which corresponds to the boundary between Time Slices 1 and 2 (~27 Ma) of Robertson et al. (2018). We interpret normal faults bounding the sedimentary basins, and throughout the igneous basement.

### 3.2 Piezometry and Deformation Temperature From Calcite Twinning

Within calcite crystals, the most common mechanism of crystal‐plastic deformation below 400 °C is twinning along the e‐plane (e.g., Burkhard, 1993; Groshong, 1988; Turner, 1953). Twin formation depends on stress orientation and requires exceeding the critical resolved shear stress (CRSS) along one of the three e‐planes (Burkhard, 1993; Ferrill, 1998; Jamison & Spang, 1976; Lacombe & Laurent, 1996; Tullis, 1980; Wenk et al., 1987). Mechanical twins have been used by many authors during recent decades as differential stress gauge (Jamison & Spang, 1976; Rowe & Rutter, 1990; Lacombe & Laurent, 1996; Rybacki et al., 2011). The minimum CRSS necessary to produce calcite twins is 5 to 15 MPa and additionally depends on grain size, porosity, temperature and strain rate (e.g., Turner, 1953; Jamison & Spang, 1976; Tullis, 1980; Laurent et al., 2000; Passchier & Trouw, 2005).

The characteristic thickness of calcite twins from very thin (<1 μm) to thicker twins (1–5 μm) represents a function of deformation temperature and deformation mechanisms (Burkhard, 1993; Ferrill, 1991; Ferrill et al., 2004). For the description of twin types and twin morphology and the corollary deformation temperatures, we generally follow the studies by Burkhard (1993) and Ferrill et al. (2004). Thin, straight type I twins (<1 μm thick) form at <170 to 200 °C; thicker type II twins (≫1 μm) can be slightly lensoid and form at 150 to 300 °C; Type III twins are several micrometers thick, show a curved and tapered morphology, and develop at temperatures >200 °C. Type IV twins are several micrometers thick, too, have a patchy, irregular morphology, and show serrate twin boundaries related to boundary migration; these twins form at temperatures >250 °C. Generally, only Types I and II are feasible for paleostrain and related stress assessment (e.g., Burkhard, 1993; Ferrill et al., 2004; Rowe & Rutter, 1990).

The evaluation of differential stresses from calcite twin densities generally follows the methods described by Brandstätter et al. (2017). The density of deformation twins (i.e., number of twins per mm) can be used to estimate differential stresses (e.g., Ferrill et al., 2004; Friedman & Heard, 1974; Rowe & Rutter, 1990; Rybacki et al., 2011). Thin sections of thirteen representative samples hosting twinned grains were analyzed for twin width and twin density using a Keyence VHX‐6000 digital photomicroscope and associated data analysis software. The mean twin width was determined from the sum of twin widths for each twin set of a calcite grain. The twin density was derived by counting the number of twins per grain, normalized to a unit length of 1 mm. The mean twin density value of each grain was used for the piezometry calculations.

Differential stresses (Δ*σ*) were calculated by using the experimentally calibrated twin density piezometer after Rybacki et al. (2011), feasible for temperatures between 20 and 350 °C:

(1) $$\Delta \sigma =10^{1.29\pm 0.02}{\rho_{\text{twin}}}^{0.50\pm 0.05}$$

Results are given in megapascals, and *ρ*_twin_ denotes the twin density (number of twins per millimeter).

Other piezometers, following the equations after Rowe and Rutter (1990), were not considered as these are very sensitive to small changes in twin density and therefore may not be suitable for application to naturally deformed rocks (for details, see Brandstätter et al., 2017).

### 3.3 EBSD Analysis of Vein Calcite

Crystallographic orientations of calcite grains in highly polished, oriented *X*‐*Z* thin sections (with *Z* parallel to the drill core axis) were measured using a scanning electron microscope which was equipped with an electron back‐scatter diffraction (EBSD) system (HITACHI S‐3400N Type II with HKL Channel5) at Shizuoka University. Twenty kilovolt accelerating voltage and low vacuum mode (30 Pa) were used. For microstructural observations and analyses, 30 μm thick thin sections were polished using 1 μm diamond paste and colloidal silica for >5 hr. Phase maps were obtained using step sizes of 5 to 7 μm. HKL Channel5 software was used to process map data by removing single pixels that differed by >10° and extrapolating nonindexed pixels with the average orientation of neighboring pixels.

EBSD data were processed using the OIM Analysis software. For the determination of potential slip systems, misorientation axes for misorientation angles from 2° to 5°, 5 to 10°, and 10° to 15° were displayed as contoured inverse pole figures (IPF) with reference to the trigonal calcite crystal system (hexagonal scalenohedral crystal class) using the MATLAB© toolbox MTEX (Bachmann et al., 2010). For two default orientations (grains, subgrains, crystals, and crystallographic axes), the misorientation is the rotation required to rotate one set of crystal axes orientation into coincidence with the other (based on a fixed reference frame) (Zhao & Adams, 1988). Misorientation axes generally are crystallographically controlled, as misorientations (e.g., subgrain boundaries) result from intracrystalline deformation (e.g., dislocation glide) by the activation of distinct slip systems. IPFs display the orientation of crystallographic axes or normal to a crystallographic plane relative to a reference axis. In this study, the reference axis is the drill core axis. A preferred orientation of distinct crystallographic axes and/or planes therefore indicates which slip systems, defined by a crystallographic plane and a misorientation axis, were activated during intracrystalline deformation.

### 3.4 Raman Spectroscopy

Raman spectra of minerals were carried out in confocal mode using a Jobin Yvon LabRam HR800 microspectrometer equipped with an Olympus BX41 optical microscope and a Si‐based charged‐coupled device detector at the NAWI Graz Geocenter, Institute of Earth Sciences, University of Graz. The instrumentation uses a 100 mW Nd‐YAG laser (532 nm emission), a grating of 1,800 grooves/mm, and a slit width of 100 μm. The spectral acquisition time was set to 10–20 s for all measurements between 100 and 1,200 cm^−1^.

## 4 IBM Forearc and Sediment Basin Structure

Despite the known differences in the mineralogical and geochemical composition of the magmatic basement, the IBM forearc crust structure is continuous from the Bonin Ridge to the trench, with changes in thickness but only minor changes in seismic wave velocity (Christeson et al., 2016; Takahashi et al., 2009). Sites U1440 and 1441 are located within small normal and oblique‐slip/strike‐slip fault‐controlled basins, respectively, on the lower forearc slope at water depths of 4,447 and 4,775 m (Reagan et al., 2015; Robertson et al., 2018). The faults extend toward the trench axis (Kurz et al., 2015; Reagan et al., 2015; Robertson et al., 2018). Sites U1439 and U1442 were drilled on the upper forearc slope at water depths of 3,128 and 3,162 m (Reagan et al., 2015; Robertson et al., 2018).

### 4.1 Upper Slope Sites

The upper slope sites are located in an area of NW‐SE trending asymmetric half‐graben structures that embody fault‐controlled, >2 km wide basins (Figure 2). Bathymetric ridges that are bounded by west dipping normal faults separate these half‐grabens (Figure 2). Normal faults extend from the seafloor to depths of at least a few hundred meters into the magmatic basement and are prevalent near all of the Expedition 352 drill sites (Christeson et al., 2016). High‐angle faults are clearly indicated by visibly disrupted layering in the upper 200 m of the basement. Dipping reflectivity coincides with many of the fault zones identified from core samples in Hole U1439C (Christeson et al., 2016; Expedition 352 Scientists, 2014) (Figure 2). Dip angles of confining normal faults are approximately 40° with antithetic normal faults and uplifted local horst ridges (Figure 2).

The seismic reflection images and the shipboard data show that the sedimentary lamination from 0 to approximately 127 m bsf at Site U1439 is layered (sub) horizontally, whereas the layers between 127 and 153 m bsf have dip angles between 10° and 14° (Reagan et al., 2015). This corresponds to the apparent dip direction displayed by seismic prestack time migrated images (Figure 2). From 153 m bsf down to the contact with the igneous basement, dip angles range from 15° to 20° (Reagan et al., 2015). The sedimentary layering at Site U1442 generally dips gently eastward down to 75 m bsf. From 75 m bsf to the basement contact dip angles continuously increase up to 35° (Reagan et al., 2015).

Displacement along the confining normal faults can be calculated from the dip angles of the faults and the sedimentary layering, taking account of the maximum sediment thickness in each basin above the hanging wall cutoff, as indicated in Figure 3. Assumptions are that the faults are planar at the scale of the cross section and that before rotating, layering was initially subhorizontal and that the faults have the same orientation and dip. Parameters are as follows: a fault dip angle *φ* of ~40° near CDP 66400 in Figure 2, an average bedding dip angle *θ* of 10° from shipboard measurements of sedimentary bedding planes, and a maximum sediment thickness x of 200 m near common depth point (CDP) 66375. Using the trigonometry displayed in Figure 3a (*d* = *x*/sin*φ*), we estimate that the fault displacement *d* is approximately 311 m.

Extension (elongation) (*e*) and the stretch factor *β* can also be calculated from the fault dip angle *φ* and bedding dip angle *θ*, using the following equations:

(2) $$e=\frac{d\cos\varphi +\mathrm{lo}*\cos\theta -\mathrm{lo}}{\mathrm{lo}}=\frac{d}{\mathrm{lo}}\cos\varphi +\cos\theta -1$$

(3) $$e\;=\;\frac{\sin\theta \cos\varphi }{\sin\theta }+\cos\theta -1=\frac{\sin\theta \cos\varphi +\cos\theta \sin\varphi }{\sin\varphi }-1\;=\;\frac{\sin\left(\theta +\varphi \right)}{\sin\varphi }-1$$

(4) $$\beta =e+1=\frac{\sin\theta \cos\varphi }{\sin\theta }+\text{cosθ}=\frac{\sin\theta \cos\varphi +\cos\theta \sin\varphi }{\sin\varphi }=\frac{\sin\left(\theta +\varphi \right)}{\sin\varphi }$$

Using a fault dip angle of 40° results in an extension *e* of approximately 0.19 for the basin adjacent to Site U1439, that is, a stretch factor *β* of approximately 1.19. Any possible strike‐slip component was not considered, as the shipboard fault and slickenside data display major normal sense of shear (Reagan et al., 2015). The elongation for the basin around Site U1442 was determined similarly. A fault dip angle *φ* of ~39° near CDP 66700 was measured in Figure 2, as well as a bedding dip angle *θ* of 8°; this results in an east‐west directed extension *e* of ~0.16, and a stretch factor *β* 1.16. The bulk extension for the upslope Sites U1439 and U1442 therefore is in the range of 0.175.

The faulting‐related strain rates can be estimated from the overall displacement, the stretching factor, and the biostratigraphic constraints. As described above, the finite bulk stretching factor calculated for the basins at Sites U1439 and U1442 is about 1.175 (*e* = 0.175). Assuming continuous fault slip over the full biostratigraphically documented time range of approximately 35 Ma gives a minimum strain rate estimate in the range of 1.58 × 10^−16^ s^−1^. An assumption of bulk fault slip between 35 and 23 Ma (Time Slice 1 as defined by Robertson et al., 2018), that is, within 12 Ma from beginning syntectonic sedimentation at 35 to the 23 Ma unconformity, gives a strain rate estimate in the range of 4.62 × 10^−16^ s^−1^.

### 4.2 Lower Slope Sites

The sedimentary basins at Sites U1440 and U1441 have an approximately symmetric graben geometry. The lateral distance between these two basins is approximately 1.5 km (see Reagan et al., 2015). The fault pattern at Site U1440 is quite irregular, whereas the basin boundaries at Site U1441 are well defined by east and west dipping bounding faults. A fault dip angle *φ*_1_ of ~32.5° near CDP 5450 was measured in Figure 2 for the limiting fault at the western basin margin; at the eastern basin margin the fault dip angle *φ*_2_ is ~33° near CDP 5200. The sedimentary layering is generally subhorizontal; in the depocenter, particularly toward the eastern basin margin, the sedimentary bedding is inclined, indicating synsedimentary/postsedimentary faulting and tilting. The bulk basin width is ~1,300 m (Figure 2c). Dip‐slip displacements can be calculated from the fault dip angles and the depth, *z*, of the base of the sedimentary succession above the igneous basement, as shown in Figure 3b; the latter (z) is in the range of ~285 m bsf. Accordingly, the dip slip displacement is in the range of 528 m (d_1_) and 520 m (d_2_) for the west and east bounding fault, respectively. The related overall extension is therefore given by the ratio of the sum of the extensions on each fault (Δ*l*_1, 2_), divided by the original sediment bed length (*l*_0_) (Figure 3b), with Δ*l*_1_, Δ*l*_2_, and *l*_0_ being in the range of 538, 416, and 261 m, respectively. This results in an extension *e* of ~3.655. Assuming continuous fault slip over the full biostratigraphically documented time range of approximately 35 Ma gives a minimum strain rate estimate in the range of 3.31 × 10^−15^ s^−1^ without consideration of any potential strike slip component. An assumption of bulk fault slip between 35 and 23 Ma (Times Slice 1 as defined by Robertson et al., 2018) gives a strain rate estimate in the range of 9.66 × 10^−15^ s^−1^.

The calculation using tilted blocks (upper slope sites) yields stretching of the crust in the entire block‐faulted area in terms of regional extension. The calculation in the symmetric graben (lower slope sites), however, yields the stretching of the graben itself and is therefore very local. The regional stretching basically depends on how far away the next graben is from the graben under consideration. The lateral distance from the eastern margin of Site U1441 basin to the western margin of Site U1440 basin is approximately 1.5 km. In order to make the stretching factor at upper slope sites and lower slope sites comparable, half of the distance from Site U1440 to the next graben on both sides (approximately 1.5 km in total), were added to *l*_0_ used in the calculation above (*l*_0_ = 261 m; *l*_0 regional_ = *l*_0_ + 1.5 km = ~1,750 m). The regional extension for the lower slope sites is therefore estimated from Δ*l*_1_, Δ*l*_2_ and *l*_0regional_, being in the range of 538, 416, and ~1,750 m. This results in a regional extension *e*_regional_ of ~0.545. Assuming continuous fault slip over the full biostratigraphically documented time range of ~35 Ma gives a minimum strain rate estimate in the range of 4.395 × 10^−16^ s^−1^. An assumption of bulk fault slip between 35 and 23 Ma (Times Slice 1 as defined by Robertson et al., 2018) gives a strain rate estimate in the range of 1.43 × 10^−15^ s^−1^.

## 5 Faults and Fault‐Related Structures

**Figure 4:**
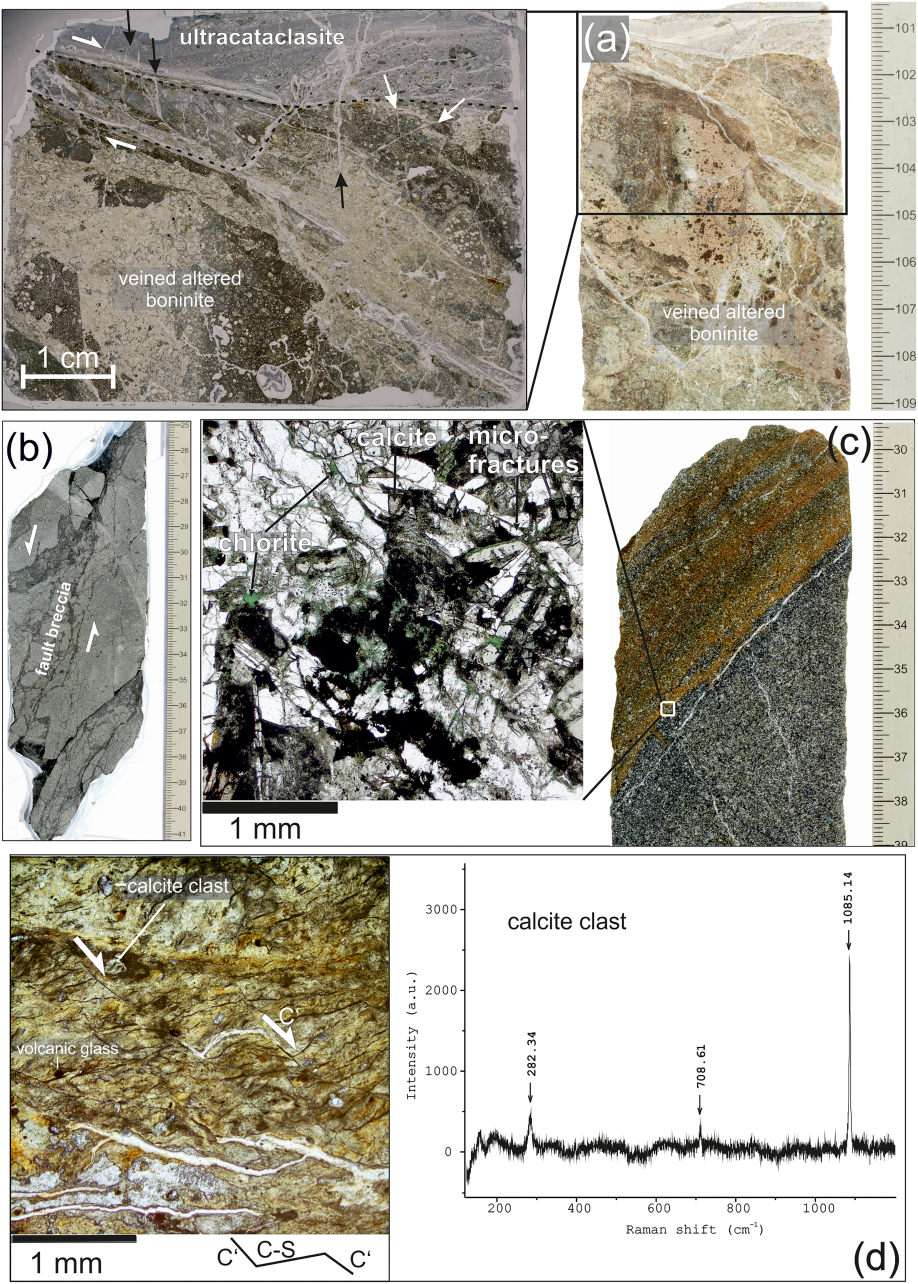
(a) Highly altered boninite, transected by an ultracataclastic shear zone and by subvertical calcite veins (marked by black arrows), with mineralized hybrid fractures (white arrows); IODP Expedition 352, Hole U1439C, Core 27R, Section 1A, 100–118 cm. (b) Cataclastic fault with fault breccia; top down displacement (normal sense of shear); IODP Expedition 352‐U1439C‐43R‐1A‐25‐41 cm. (c) Foliated alteration zone (brownish) within FAB, characterized by microfracturing, microbrecciation, and formation of secondary chlorite, calcite, clay minerals and opaque phases (microphotograph with parallel polarizers); IODP Expedition352, Hole U1440B, Core 35R, Section 1, 29–39 cm; and (d) Microphotograph (parallel polarizers) of ultramylonitic, semiductile to brittle shear zone; the ultramylonite consists of kryptocrystalline calcium carbonate with single clasts of calcite (with related Raman spectrum) and brownish amorphic volcanic glass; C′ shear bands indicate top down (normal) sense of shear; IODP Expedition352‐ U1441A‐20R‐1‐16‐18 cm. Figures 4a–4c are cutouts from shipboard core close‐up images taken by Tim Fulton (provided by International Ocean Discovery Program [IODP] and *JOIDES Resolution* Science Operator [JRSO]).

**Figure 5:**
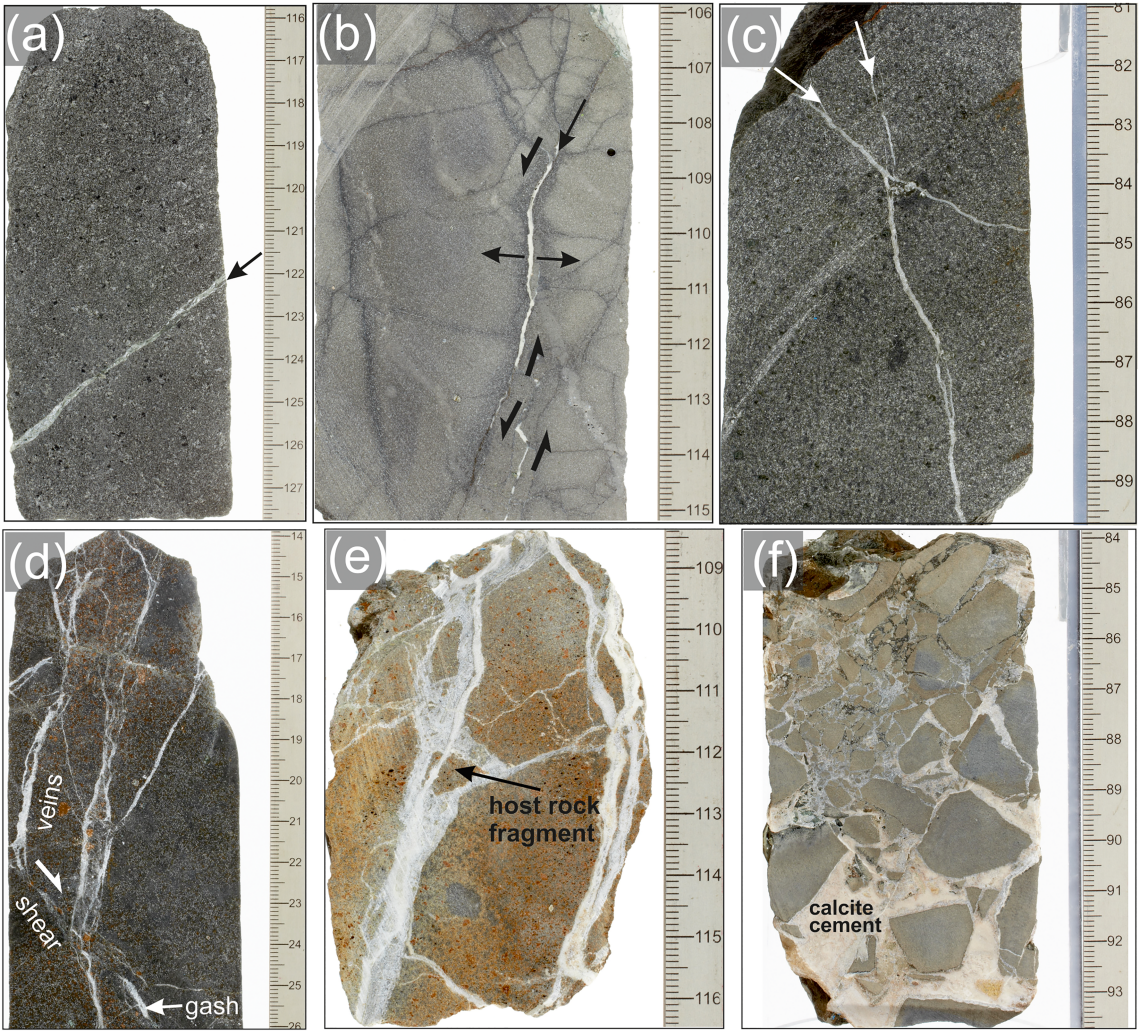
(a) Single calcite vein (marked by arrow); IODP Expedition 352, Hole U1440B, Core36R, Section 1, 116–128 cm. (b) Fragmented boninite with multiple cracks accompanied by dark alteration seams, and a single tapered calcite vein (marked by arrow); this opened as a tension gash along releasing bend with top down kinematics; IODP Expedition 352‐U1442A‐30R‐3A‐106‐115 cm. (c) Conjugate hybride fractures (marked by arrows) within boninite, filled with calcite; IODP Expedition 352‐U1439C‐41R‐1A‐81‐90 cm. (d) Horsetail calcite veins and splays associated with distinct single sets of shear fractures with top down displacement within boninite; the discrete tension gashes were formed along shear fractures; IODP Expedition 352‐U1439C‐23R‐2W‐14‐26 cm. (e) Complex multiple carbonate vein network resulting from hydrofracturing within altered boninite; discrete boninite fragments (marked by arrow) are embedded in vein precipitates; IODP Expedition 352‐U1439C‐4R‐1A‐108‐117 cm. (f) Hydraulic breccia of Forearc Basalt (FAB), cemented by calcite precipitate; IODP Expedition 352‐ U1440B‐19R‐1A‐84‐94 cm. Figures are cutouts from shipboard core close‐up images taken by Tim Fulton; (provided by International Ocean Discovery Program [IODP] and *JOIDES Resolution* Science Operator [JRSO]).

Several of the IBM sites revealed fault zones with various kinematic features, including discrete faults and cataclastic shear zones (Figure 4), together with extensional fractures and veins with minerals that were mainly precipitated from hydrothermal fluids (Figure 5). Sites U1439 and U1441 are situated close to the related sediment basin axis, whereas Site U1442 is situated on a topographic, uplifted basement ridge. Drilling at these sites penetrated several minor fault zones within the igneous basement, most of these being related to the major, basin‐bounding normal, oblique, and strike‐slip faults. Site U1440 is located off the sedimentary basin axis, and penetrated the normal fault terminating this basin to the west, as well as related secondary extensional structures.

Fault structures that were observed in the Expedition 352 forearc sites are documented in detail by Reagan et al. (2015) and are summarized below. These data are supplemented by postcruise microstructural analyses.

### 5.1 Upper Slope Sites

#### 5.1.1 Site U1439

Hole 1439C revealed cataclastic fault zones from ~348 to 401 m bsf (fault zone 1), from ~420 to 446 m bsf (fault zone 2), and from ~475 to 535 m bsf (fault zone 3). The fault zones have variable thickness in the range of a few centimeters to decimeters and are characterized by continuous downward loss of cohesion. Extensional structures, including fractures and normal‐sense slickensides, tend to offset reverse faults.

An ultracataclasitic shear zone (Fault Zone 1) with host rock fragments of millimeter to centimeter size is transected by subvertical veins with an en echelon geometry indicating normal sense of shear (Figure 4a). The adjacent host rock (boninite) is highly altered and bleached (Figure 4a); veins are abundant between 348.8 and 359.8 m bsf.

Fault rocks within Fault Zone 2 comprise cohesive fault breccia with millimeter to centimeter sized host rock fragments embedded within a fine‐grained matrix (<0.2 mm grain size) (Figure 5f). The matrix amounts to less than 20%.

Fault Zone 3 contains slightly cohesive to incohesive fault breccia (centimeter‐sized fragments) and cataclasites, with friable centimeter sized fragments of host rock material, partly surrounded by fine grained fault gouge and an irregular fracture network. Discrete cataclastic shear bands comprise incohesive cataclasite and fault gouge. Vein quantity and vein thickness decrease remarkably downward within Fault Zone 3. Veins are almost missing below 515 m bsf. On the other hand, the abundance of slickensides and shear fractures increases. A domain of slightly cohesive fault breccia (centimeter‐sized fragments) was recovered at from ~524 to 529.39. This deepest fault zone caused structural instability and prevented deepening of Hole U1439C. Cataclastic shear zones with feasible recovery generally indicate top‐down kinematics (Figure 4b).

#### 5.1.2 Site U1442

Hole U1442A revealed fault zones from 238.20 to 267.45 m bsf (Fault Zone 1), from 432.80 to 444.80 m bsf (Fault Zone 2), and from 490.90 to 502.20 m bsf (Fault Zone 3).

Fault Zone 1 comprises medium‐ to coarse‐grained cataclasites and fault breccias and cohesive, foliated fault gouges, forming a ~40 cm wide phyllonitic shear zone between 248.21 and 248.59 m bsf. Single sets of shear bands indicate normal sense of shear.

Fault Zones 2 and 3 are basically characterized by single cataclastic shear zones. The shear zone thicknesses range from a few to 15 cm. Fault rocks comprise fault breccias with host rock fragments, up to 1 cm in diameter, as well as slightly cohesive, fine‐grained cataclasites with single millimeter‐sized host rock fragments and fault gouges. The fault damage zones comprise steeply dipping to subvertical multiple sets of slickensides with predominant normal to oblique‐normal slip.

### 5.2 Lower Slope Sites

#### 5.2.1 Site U1440

Extensional fractures, generally with inclined to subvertical orientations, are the main structural features from 144.76 to 183.50 m bsf. Subvertical to inclined mineralized veins, up to 7 mm thick, are steeply dipping to subvertical. Locally, these veins occur as conjugate sets, forming a vein network (Figures 5c and 5e).

Macroscopic, centimeter‐thick cataclastic shear zones (Figure 4c) occur between 145.00 and 146.00 m bsf, 281.00 and 291.00 m bsf, and 358.00 and 369.00 m bsf. The shear zones are characterized by microbrecciation and by a macroscopic foliation defined by (sub) parallel fracture sets and clusters of chlorite. Plagioclase and pyroxenes show intense microfracturing; microfractures are partly filled by chlorite, calcite, and/or clay minerals.

#### 5.2.2 Site U1441

The general structure at Site U1441 is highly irregular at the sediment‐igneous basement boundary and along the basin margins and is characterized by multiple minor faults and fracture zones. Fault damage zones comprise steeply dipping to subvertical multiple sets of slickensides with predominant left‐lateral to oblique‐normal left‐lateral shear. Mineralized veins are steeply dipping to subvertical.

The lowermost interval of Hole U1441A (~180 m bsf) revealed a semiductile low‐angle shear zone (Figure 4d). This shear zone is characterized by an ultramylonitic fabric with distinct carbonate clasts and clasts of amorphic volcanic glass embedded within an ultrafine‐grained, sheared calcium carbonate matrix, as revealed by Raman Spectroscopy. Clasts reach up to 500 μm in size. Shear bands appear as subparallel sets, indicating top‐down sense of shear (Figure 4d). The shear bands are transected by subparallel sets of inclined shear fractures, indicating normal displacement, too. This shear zone marks the contact between clinopyroxene‐phyric basalt above and aphyric to sparsely clinopyroxene‐bearing basalt below (see the geochemical stratigraphy described by Reagan et al., 2015; Shervais et al., 2019). FAB pieces that were recovered below and above this shear zone do not indicate comparable deformation or alteration.

### 5.3 Expedition 351 Site U1438 (ASB)

Pervasive faulting is not documented at Site 351‐U1438 (Arculus, Ishizuka, Bogus, & the Expedition 351 Scientists, 2015), as it is located centrally within the ASB. Seismic reflection images (see Arculus, Ishizuka, Bogus, & the Expedition 351 Scientists, 2015) display almost horizontally layered sediment beds and an almost unfaulted basement. Minor faults with reverse sense of shear are locally documented within the sedimentary cover. The core images from the magmatic basement do not display pervasive fracturing, neither. Fractures usually occur as discrete, single fractures. Veins are not as abundant as at Sites U1439 to U1442 and generally occur as subvertical, millimeter‐thick single veins filled with calcite and zeolite.

## 6 Veins and Wall Rock Alteration

Vein structures and alteration textures that were observed during IODP Expedition 352 are documented in detail by Reagan et al. (2015) and are summarized below. These data are supplemented by postcruise microstructural and piezometry analyses.

### 6.1 Macroscale Vein Structures

Mineralized veins (Figure 5) were observed at several of the IBM sites and comprise tension fractures, hybrid (tension and shear) fractures, tension gashes related to releasing bends and extensional step‐overs that are distributed along distinct shear fractures. The vein dip angles are usually >45°. In general, the individual drill sites are not particularly characterized by any distinct vein type. Veins occur as single subvertical features (Figure 5a), single bent and tapered tension gashes (Figure 5b), or as conjugate hybrid mineralized fractures (Figure 5c). Some of these veins, like at Site U1440, occur as intersecting networks (Figure 5c). The related vein dip angles (from ~25° to 50°, and from ~65° to 90°, respectively) form two clusters. Other vein types are splayed extensional veins with horsetail arrangement, associated with distinct single sets of shear fractures with top‐down displacement (normal sense of shear) (Figure 5d), or as complex multiple vein networks, resulting from hydrofracturing (Figure 5e). In addition, distinct domains with cataclasites and fault‐related hydraulic breccias comprise disintegrated host rock fragments that are cemented by mainly sparitic carbonate (Figure 5f).

Calcite, various zeolites, and clay minerals are the main vein fillings. Vein thicknesses vary from <1 to 15 mm. Calcite veins are usually filled with crystalline blocky (Mg‐) calcite. A few wider (>15 mm) veins have antitaxial, zoned calcite fiber mineralizations that indicate incremental steps of extension and precipitation.

### 6.2 Vein Calcite Microstructures and Twin Density Piezometry

**Figure 6:**
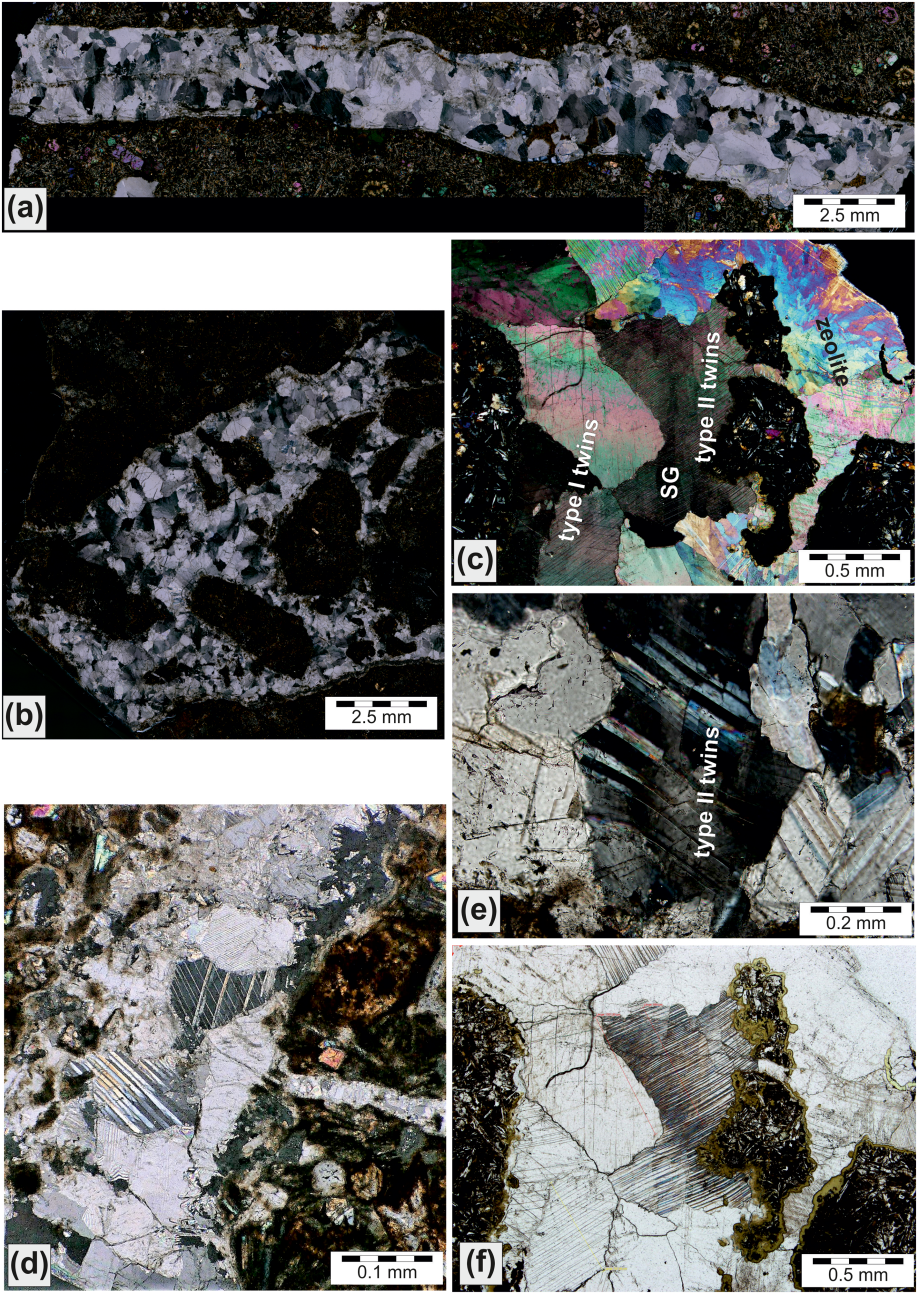
Microphotographs (crossed polarizers) showing representative vein microstructures: (a) Blocky calcite vein within boninite; average calcite grain size is 0.7 mm; Sample 352‐U1439C‐26R‐2‐W 9/11‐KURZ. (b) Irregular blocky calcite vein with boninite fragments embedded in vein minerals; average calcite grain size is 0.3 mm; Sample 352‐U1439C‐29R‐4‐W 60/63‐KURZ. (c) Blocky calcite with thin type I and thicker Type II twins and subgrain (SG) formation; Sample 351‐U1438E‐79R‐2‐W 111/114. (d) Blocky calcite vein within boninite with thick Type II twins; Sample 352‐U1439C‐23R‐2‐W 15/21‐KURZ. (e) Bent and tapered Type II twins; calcite host shows undulatory extinction; Sample 352‐U1439C‐26R‐2‐W 9/11‐KURZ. (f) Bent Type I and II twins; calcite host shows undulatory extinction; Sample 351‐U1438E‐82R‐2‐W 43/52.

Each of the investigated host rocks contained blocky calcite veins with twinned grains. Boninites, FABs and samples from ASB hosted nine, two, and three veins, respectively, that were suitable for twin density measurements. The grains predominantly display one twin set, but locally two sets and rarely three sets. Following the classification of Burkhard (1993), almost 90% of twins are of Type II, with subordinate examples of Type I. Grains hosting Type I twins tend to show higher twin densities than those with Type II twins. The apparent width of the Type II twins ranges from 2 to 45 μm. Some twins are bent or show tapered endings (Figure 6). Locally, offsets along intracrystalline microfractures are observed.

**Figure 7:**
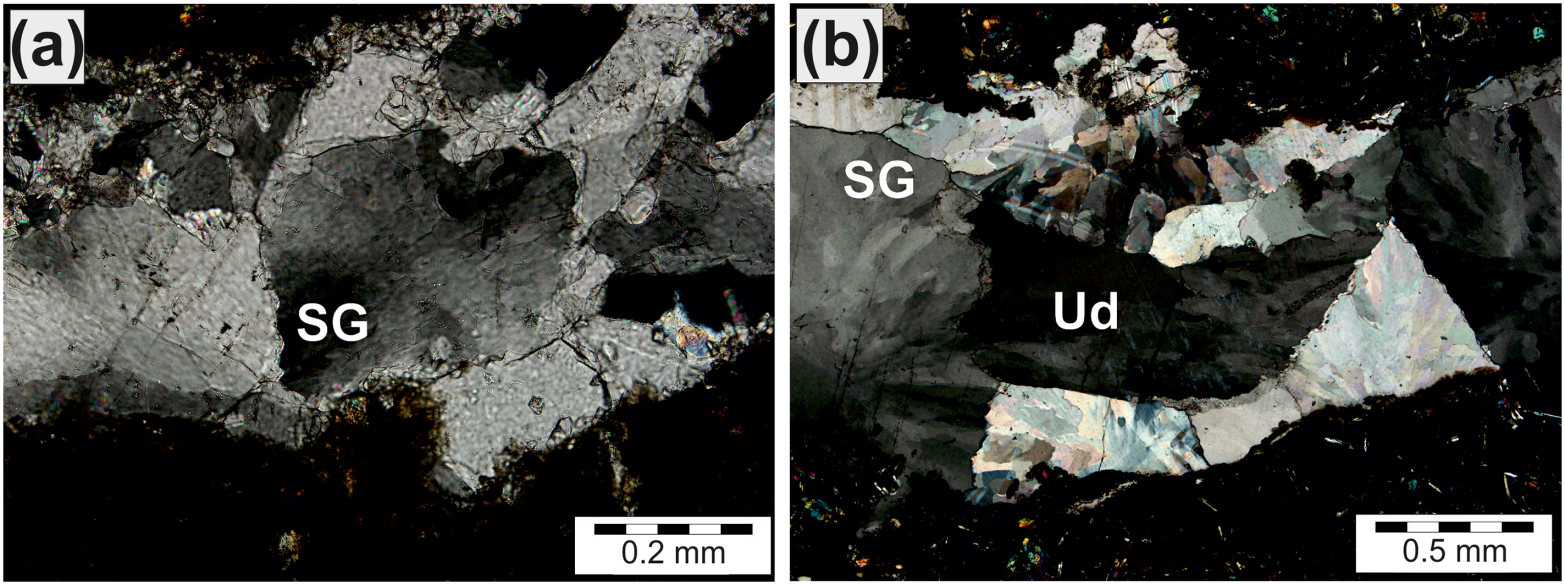
Microphotographs (crossed polarizers) showing representative vein microstructures: (a) Subgrains (SG) within blocky calcite; average subgrain size is 0.15 mm; Sample 352‐U1439C‐26R‐1‐W 110/120‐KURZ. (b) Undulatory (Ud) extinction of calcite and subgrains (SG) within blocky calcite; average subgrain size is 0.1 mm; Sample 351‐U1438E‐79R‐2‐W 111/114.

According to Burkhard (1993), Type II twins point to deformation temperatures between ~150 and 300 °C. While tapered twins are distinctive of deformation and distinguish from growth twins, bent twins as well as offsets along microfractures point to advanced intracrystalline deformation. Moreover, undulatory extinction and the formation of subgrains within calcite grains with tapered and curved twins (Figure 7) indicate intracrystalline, plastic deformation mechanisms (dislocation glide and dislocation creep) (e.g., Twiss, 1977; Wheeler et al., 2001).

**Figure 8:**
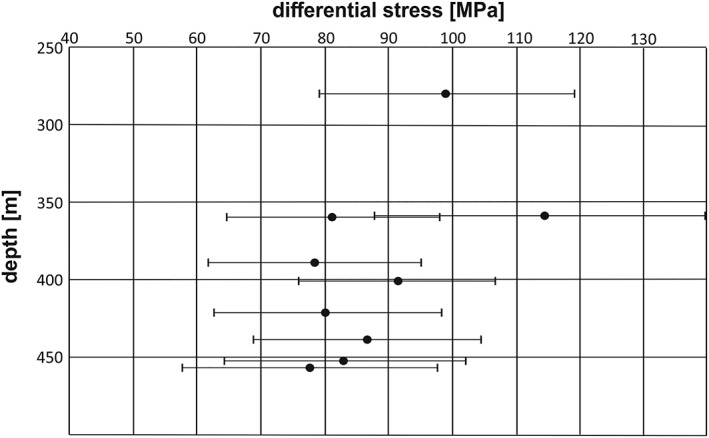
Differential stress data after Rybacki et al. (2011) versus borehole depth for site U1439C; error bars indicate 2*σ* standard deviation.

Most of the detailed studies were performed on samples from the boninite sites (U1439 and U1442), mainly due to a higher vein recovery. Differential stresses at the boninite sites range from 34 to 150 MPa with an average of 88 ± 9 MPa (Table 2). Veins from U1440 and U1441 (FAB) and from Site U1438 (ASB) show average differential stresses of 69 ± 7 (39 to 87 MPa) and 91 ± 33 MPa (34 to 181 MPa), respectively (Table 2). Basically, no correlation of differential stresses with depth was observed (Figure 8).

**Table 2:** Results of Piezometric Analyses After Rybacki et al. (2011)

Sample	# grains	Twin sets	*Twin type*		# twins mm^−1^	Δ*σ* [MPa]
BON‐1	6	1	II > I	range; average	17–50; 27 ± 9	80–138; 99 ± 22
BON‐2	13	1 > 2	II ~ I	range; average	10–59; 36 ± 13	62–150; 114 ± 26
BON‐3	16	1 > 2	II	range; average	9–33; 18 ± 6	58–112; 81 ± 17
BON‐4	15	1 > 2	II	range; average	7–36; 17 ± 6	52–117; 78 ± 17
BON‐6	13	1 > 2	II	range; average	12–42; 23 ± 6	68–126; 91 ± 16
BON‐7	17	1 > 2 > 3	II	range; average	9–40; 17 ± 17	58–123; 80 ± 18
BON‐8	31	1 > 2	II > I	range; average	10–35; 21 ± 7	62–115; 87 ± 18
BON‐9	19	1 > 2	II > I	range; average	6–45; 19 ± 19	48–131; 83 ± 19
BON‐10	17	1 > 2	II > I	range; average	3–54; 17 ± 7	34–143; 78 ± 22
FAB‐1	1	1	II	range; average	15; 15	76; 76
FAB‐2	7	1 > 2	II	range; average	4–20; 10 ± 3	39–87; 62 ± 8
ASB‐1	13	1 > 2	II	range; average	4–41; 18 ± 9	39–125; 80 ± 19
ASB‐2	10	1 > 2	II	range; average	3–14; 7 ± 2	34–73; 52 ± 9
ASB‐3	7	1 > 2	I > II	range; average	7–86; 56 ± 19	52–181; 140 ± 43

**Table 3:** Strain Rates for Different Differential Stresses and Temperatures, Calculated From the Power Law Equation of Schmid (1982) and Rutter (1995)

	T=100°C		T=125°C		T=150°C		T=200°C	
	log strain rate	strain rate	log strain rate	strain rate	log strain rate	strain rate	log strain rate	strain rate
σ=35 Mpa	‐40.202	3.471 e‐18	‐37.44	5.495 e‐17	‐35.05	6.27 e‐16	‐30.906	3.78 e‐14
σ=50 Mpa	‐39.272	8.798 e‐18	‐36.51	1.39 e‐16	‐34.075	1.59 e‐15	‐29.977	9.575 e‐14
σ=75 Mpa	‐38.216	2.527 e‐17	‐35.454	4.00 e‐16	‐33.019	4.57 e‐15	‐28.92	2.75 e‐13
σ=80 Mpa	‐38.047	2.995 e‐17	‐35.286	4.73 e‐16	‐32.851	5.407 e‐15	‐28.752	3.26 e‐13
σ=100 Mpa	‐37.466	5.35 e‐17	‐34.704	8.477 e‐16	‐32.269	9.677 e‐15	‐28.171	5.827 e‐13
σ=140 Mpa	‐36.589	1.827 e‐16	‐33.828	2.035 e‐15	‐31.392	2.326 e‐14	‐27.294	1.40 e‐12

As described above, differential stresses were estimated by calcite twin density following the method of Rybacki et al. (2011), Rybacki et al., 2013). To estimate related strain rates we used the power law creep equation for calcite, as provided by Schmid (1982) and Rutter (1995):

log (strain rate) = −5.5–314 kJ/2.303 *RT* + 6.0 log*σ*

where σ = differential stress, *R* = gas constant (0.008314 kJ/mol K) and *T* = absolute temperature (°K).

The results of iterative strain rate calculations are shown in Table 3, indicating that strain rates (at given differential stresses) are highly sensitive to temperature. Depending on the assumed deformation temperatures, derived from calcite twin morphology and fault‐ and vein‐related alteration assemblages (see below), the strain rates range from 10^−18^ to 10^−16^ at 100 °C, from 10^−16^ to 10^−14^ at 150 °C, and from 10^−14^ to 10^−12^ at 200 °C.

### 6.3 Fault‐ and Vein‐Related Wall Rock Alteration

Deformation temperatures can supplementarily be assessed by wall rock alteration mineral assemblages within the fault zones and adjacent to mineralized veins. In Hole U1439C wall rock alteration is indicated by secondary clay minerals, different zeolite types, and mutable occurrence of calcite. Additionally, the presence of talc within the lower sections of Hole U1442 indicates zeolite facies (metamorphic) conditions in the range of 100° to 150 °C. The overall alteration degree is low to moderate at these sites (see Reagan et al., 2015, for details).

The extent of alteration at Site U1440 is generally low. Secondary minerals are clay minerals (montmorillonite, interlayered montmorillonite‐illite, and illite), calcite, and small amounts of zeolite (largely laumontite and phillipsite) (Reagan et al., 2015). Macroscopic foliation fabrics defined by clusters of chlorite were observed between 145.00 and 146.00 m bsf, 281.00 and 291.00 m bsf, and 358.00 and 369.00 m bsf. The alteration mineral assemblages indicate upper zeolite facies to prehnite‐pumpellyite facies metamorphic conditions. Alteration mineral assemblages at Site U1441 comprise zeolites (phillipsite, merlinoite, chabazite, and analcime), smectite group clays, and minor calcite. These assemblages indicate moderate to high alteration with a tendency to decline downhole (Reagan et al., 2015). In general, the alteration at the upslope Sites U1439 and U1442 appears to be lower, compared to the downslope sites. This difference also applies to the metamorphic conditions and the related (deformation) temperatures, allowing for higher strain rates at similar differential stresses at the downslope sites.

## 7 EBSD Analyses

The samples selected for the EBSD analysis are blocky calcite veins from Expedition 351 Site U1438 (ASB rear arc), and from Expedition 352 Sites U1439 (boninite) and U1441 (FAB). Several calcite veins contain twins and show undulatory extinction, bent twins and multiple subgrain boundaries. Twins are usually overprinted by or interact with subgrains (Figure 7).

**Figure 9:**
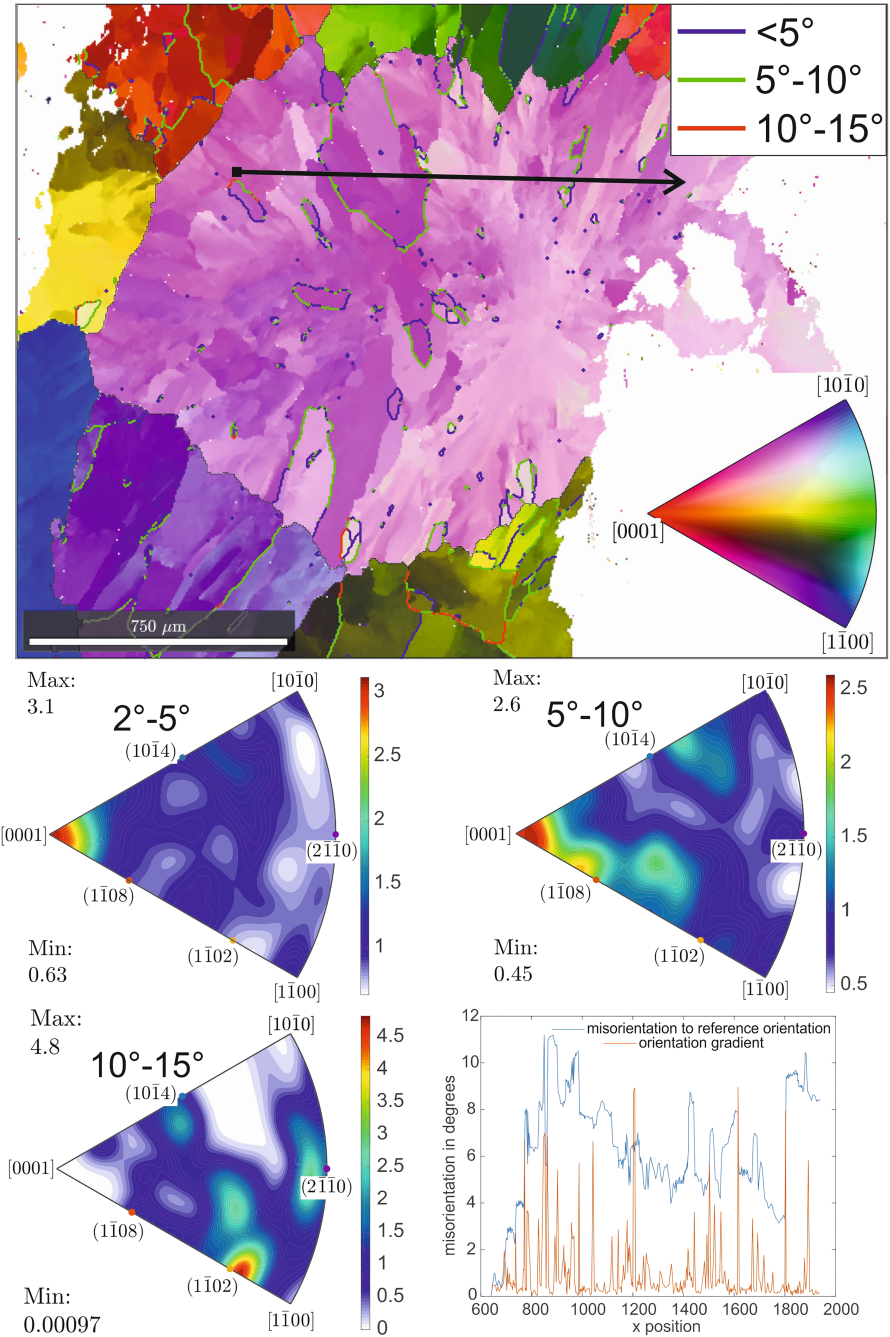
Results of EBSD mapping for Sample ASB‐3. Vein calcite contains several grains with internal subgrains. Subgrains are displayed by color gradient with a tolerance angle of 2°; the color coding of grains refers to the orientation of crystallographic axes of a hypothetical single crystal. Subgrain boundaries with misorientation angles of <5°, 5–10°, and 10–15° are marked by blue, green, and red lines, respectively. The black arrow indicates the direction of the misorientation profiles. Misorientation profile: the blue line represents point to origin and the red line point to point misorientation angles. Color‐coded map (inverse pole figure map): low angle boundaries (red and blue lines), and high angle boundaries (black line). Misorientation axes are shown in countered inverse pole figures (IPF) for misorientations of 2° to 5°, 5–10°, and 10–15°. For exact sample locations see Table 1.

**Figure 10:**
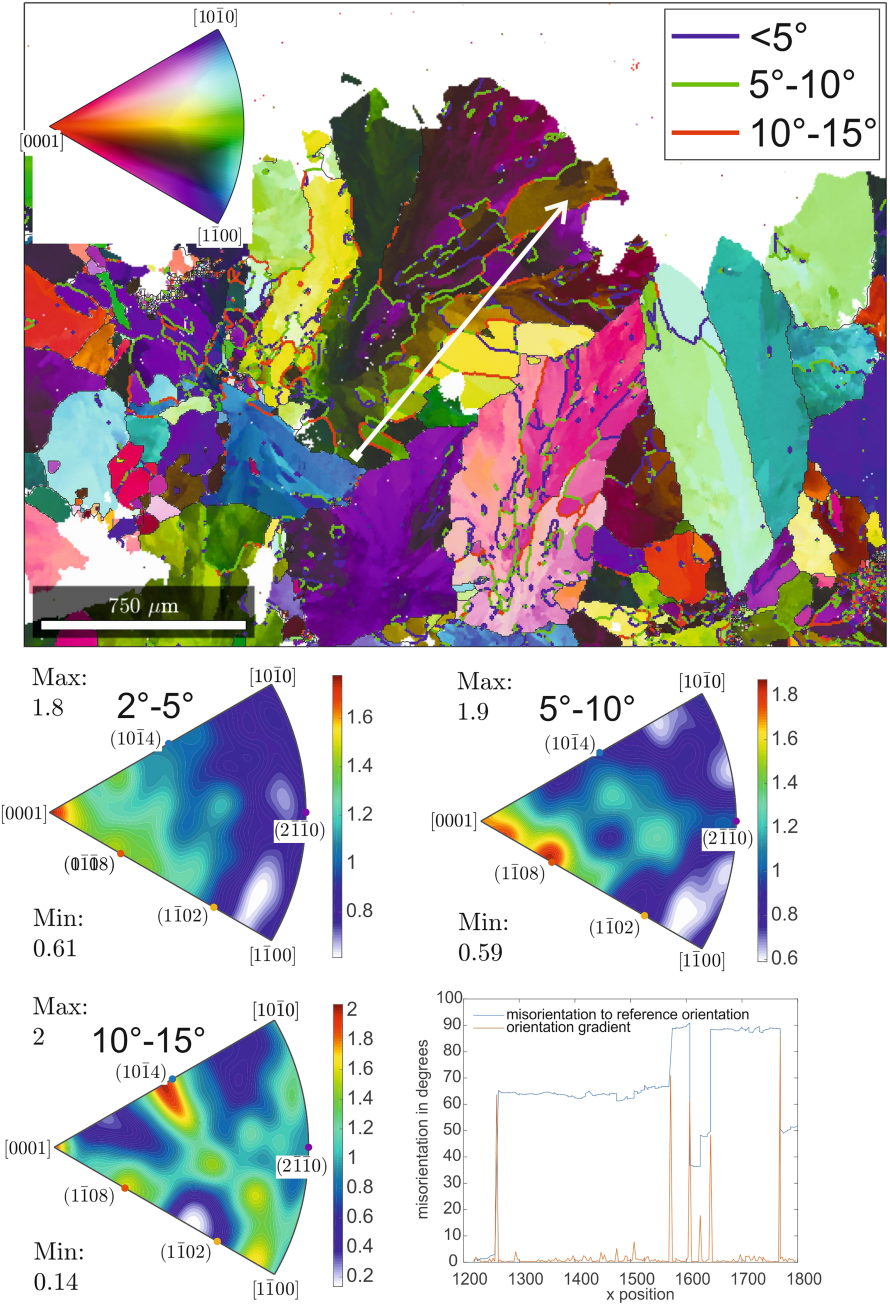
Results of EBSD mapping for Sample BON‐9. For legend and explanation see figure captions at Figure 9. For exact sample locations see Table 1.

**Figure 11:**
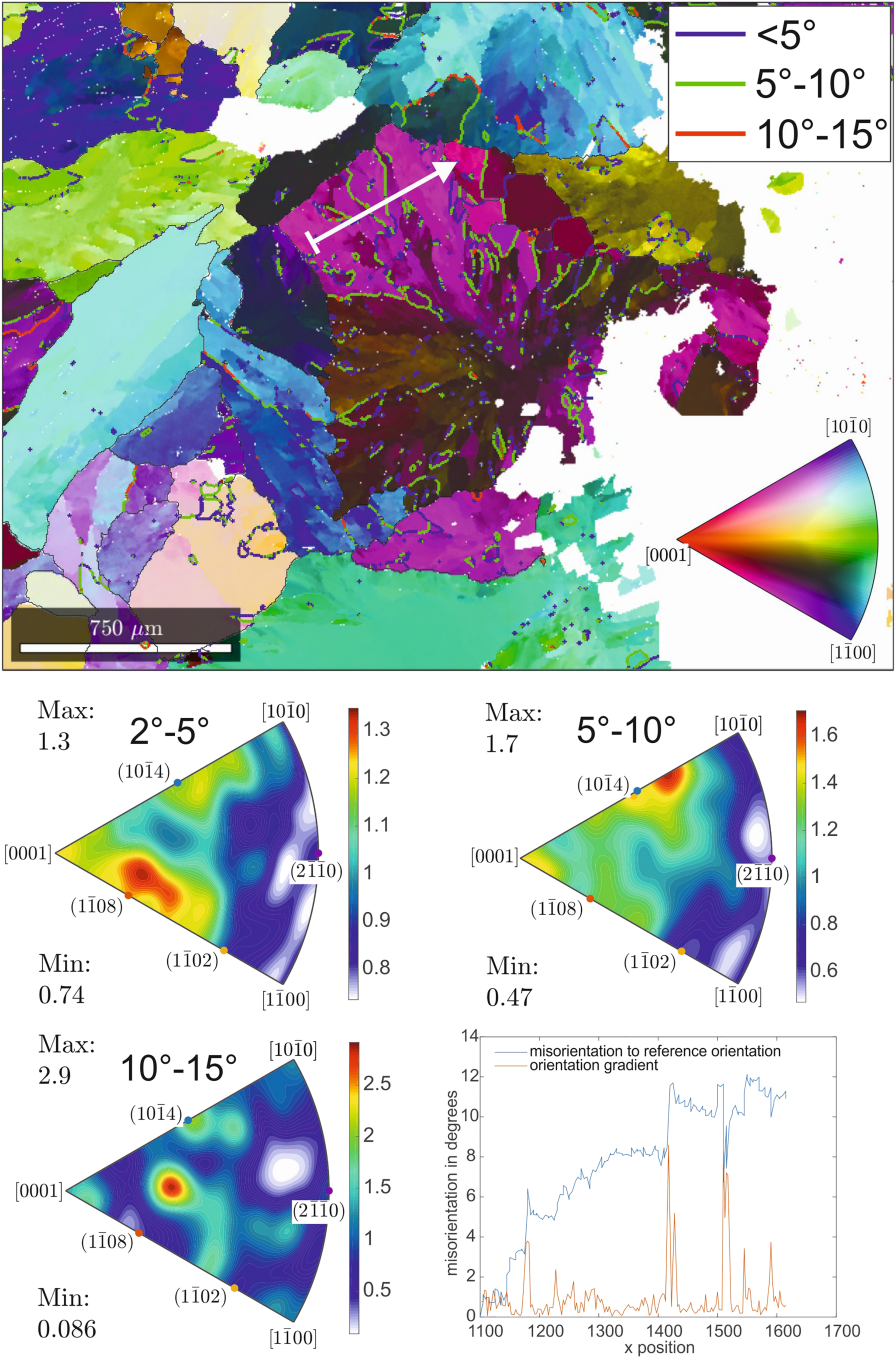
Results of EBSD mapping for Sample FAB‐3. For legend and explanation see figure captions at Figure 9. For exact sample locations see Table 1.

The subgrains within the blocky calcite grains show misorientations between 1° and 70° (Figures 9, 10, 11). The blocky calcite grain sizes range from ~ 0.5 to 2.5 mm (average > 1 mm); subgrain sizes vary between 30 und 50 μm. The misorientation profiles, as well as the EBSD maps, display misorientation gradients by color coding (Figures 9, 10, 11), visualizing distinct shifts of crystal‐lattice orientation along the subgrain boundaries.

The internal calcite microstructure within Sample ASB‐3 (Site U1438) is characterized by the formation of elongate subgrains with an average size of ~38.5 μm and an aspect ratio from 1.5 to 10 (Figure 9). Misorientation angles range from 1° to 9°; only a few subgrain boundaries show misorientation angles exceeding 5°. The misorientation profile shows higher misorientation angles in the rim areas with reference to the starting point than within the internal grain domains. Major jumps in misorientation angle are coupled with low‐angle grain boundaries. For the determination of potential slip systems, misorientation axes for misorientation angles from 2° to 5°, 5 to 10°, and 10° to 15° were displayed as IPF with reference to the trigonal calcite crystal system (hexagonal scalenohedral crystal class) (Figures 9, 10, 11). Sample ASB‐3 shows a maximum in orientation distribution function (ODF) density (max) of 3.1 and 2.6, respectively, parallel to the crystallographic c‐axis [0001] at low (2° to 5° and 5° to 10°) misorientation angles. IPF for misorientation angles from 10° to 15° are characterized by a maximum relative to one of the f‐ planes (1 $\bar{1}$02).

The calcite grains in the vein Sample BON‐9 (Site U1439) have sizes in the same range as sample ASB‐3. The subgrains are generally elongated, with aspect ratios between 1.5 and 5, and an average grain size of ~30 μm (Figure 10). The subgrains in this sample appear to be more evolved in comparison to Sample ASB‐3, with misorientation angles of up to 90° and the formation of discrete high‐angle boundaries. The misorientation profile shows several domains of internally constant misorientation with reference to the starting point; these domains are separated by high angle misorientation boundaries. For low angles (2° to 5° and 5–10°), the IPFs show a maximum in ODF density (max) of 1.8 and 1.9, respectively, parallel to the crystallographic c‐axis [0001]. In addition, the 5° to 10° IPF is characterized by a maximum in the area of (1 $\bar{1}$ 0 8). IPF for misorientation angles from 10° to 15° are characterized by a maximum normal to one of the r‐planes (1 0 $\bar{1}$4).

The intracrystalline calcite microstructures within Sample FAB‐3 (Site U1441) do not differ significantly from the samples described above (Figure 11). Subgrains have elongate shapes with aspect ratios between 1.5 and 3; the average subgrain size is ~40 μm. An incremental increase in misorientation angle is apparent along the misorientation profile (Figure 11). The internal domains are separated by subgrain boundaries with misorientation angles of 5° to 10°. For low angles (2° to 5°), the IPFs reveal a maximum in ODF density (max) of 1.3 in the area of (1 $\bar{1}$0 8), whereas the 5° to 10° IPFs are characterized by a maximum close to (1 0 $\bar{1}$4). IPF for misorientation angles from 10° to 15° are characterized by a maximum at (1 0 $\bar{1}$4) and (1 $\bar{1}$0 8).

## 8 Discussion

Seismic reflection data at the scale of the IBM forearc upper crust, mesoscale structures within drill cores, and fault and vein microstructures provide an insight into the tectonic evolution of the outer IBM forearc. The tectonic structures indicate that IBM forearc deformation essentially took place after formation of the igneous basement. Postmagmatic extension triggered the formation of asymmetric sediment basins, notably the half‐grabens at Sites 352‐U1439 and 352‐U1442 on the upper trench slope, and symmetric graben structures at Sites 352‐U1440 and 352‐U1441 closer to the trench, with localized shear along multiple sets of faults. Faulting was accompanied by syntectonic pelagic, hemipelagic, and volcaniclastic sedimentation.

### 8.1 Deformation Conditions Derived From Microstructure Analysis

Calcite twin piezometry yielded mean differential stresses in the range of 52 to 140 MPa in general (Table 2), with mean differential stresses of ~88 MPa at upslope sites, ~69 MPa at downslope sites, and ~90 MPa at Site U1438, respectively. Deformation microstructures indicate that subgrain formation and mechanical twinning have interacted, that is, subgrains overprint twins and vice versa. Subgrain formation in particular can be related to the effects of either temperature‐reduced CRSS or increased differential stresses and/or strain rates. These effects in turn permitted the activation of intracrystalline deformation mechanisms, including dislocation glide and dislocation climb (De Bresser & Spiers, 1993, 1997; Rogowitz et al., 2014).

**Figure 12:**
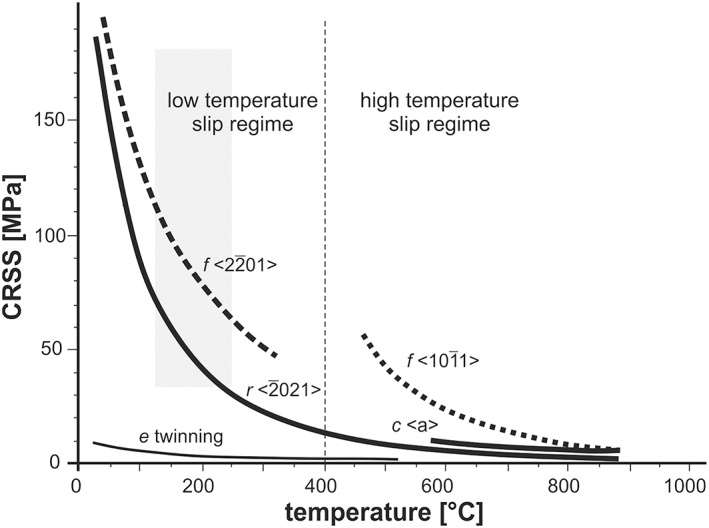
Diagram, after De Bresser and Spiers (1997) summarizing the intracrystalline deformation mechanism of calcite. The gray box displays the range of obtained differential stresses; the corresponding range of deformation temperature was acquired from different twin parameters according the classifications after Ferrill et al. (2004) and Burkhard (1993), and also the host rock alteration mineral assemblages described by Reagan et al. (2015). Mechanical e‐twinning occurs at low CRSS and at proportional low temperatures. With increasing differential stresses and/or strain rates, *f* and *r* slip and subgrain formation is enabled.

The temperature limits are constrained by the calcite twin morphology (Type I and Type II twinning), indicating deformation conditions between approximately 125 and 250 °C. The upper temperature limits are also constrained by alteration‐related mineral assemblages that indicate zeolite to prehnite‐pumpellyite metamorphic conditions. The activation of high‐temperature slip systems in calcite (Figure 12) can therefore be neglected with reference to these upper temperature limits.

The related differential stresses were derived from calcite twin piezometry (Table 2). Although calcite piezometers typically reveal a wide range of results (also in standard deviation), these differential stresses in the range of 50 to 140 MPa were by all means sufficient to activate these low‐temperature‐high‐stress slip systems. By assumption of generally constant differential stresses during bulk deformation at several sites, the inferred subgrain misorientations suggest the activation of low‐temperature slip systems, in particular the f‐ as well as the easier r‐slip system (Figure 12).

As deformation temperatures appear to be higher at the downslope sites, compared to the upslope sites (~25–50 °C) as indicated by alteration mineral assemblages), the related strain rates may also slightly increase toward the trench. The derived mean differential stresses in the range of 70 to 90 MPa and deformation temperatures of ~125 °C and ~150 °C at upslope and downslope sites, respectively, result in strain rates in the range of 4 × 10^−16^ s^−1^ at 125 °C, and 4.5–5.5 × 10^−16^ s^−1^ at 150 °C (see Table 3).

In detail, the microstructures also indicate a variation in the dominant deformation mechanisms from Site U1438 toward Site U1441. At Site U1438 (ASB), the dominant calcite deformation mechanisms are twinning and f‐slip. At Site U1439 and Site U1441, the dominant calcite deformation mechanisms are twinning and f‐slip and, in addition, r‐slip. This indicates locally changing deformation conditions from the rear arc toward the forearc, probably depending on differences in the tectonic setting, and essentially for Sites U 1439 to U 1442 on the distance to the trench. In particular, at Site U1441 extension was accommodated by a (semi) ductile shear zone (Figure 4d) along the western margin of a graben‐shaped sediment basin. The formation of chlorite at Site U1440 (Figure 4c) also shows that the alteration was accompanied by fluid infiltration and related hydration reactions. Shearing combined with these reactions therefore potentially facilitated shear zone weakening, allowing for an increase of strain rates toward the trench. This increase can, conversely, enable the activation of additional (high stress) slip systems.

### 8.2 Faulting and Formation of IBM Forearc Basins

Extensional faults and veins reveal the postmagmatic deformation within the Izu‐Bonin forearc upper crust subsequent to the formation of its magmatic basement. Differential stresses, derived from calcite microstructures, were sufficient to exceed common tensile strength of the oceanic crust. This was potentially aided by hydrothermal fluid pressures as indicated by, for example, hydraulic breccias (Figure 5f), allowing crustal failure during incipient extension. Time constraints for the tectonic deformation of the IBM forearc are provided by the magmatic ages from the igneous basement and the biostratigraphic record of the sedimentary cover. Zircon ages (Ishizuka, Tani, et al., 2011; Reagan et al., 2013; Reagan et al., 2019) and ^40^Ar/^39^Ar ages from FABs and boninites from Expedition 352 (Reagan et al., 2019) indicate an Eocene igneous basement age of 52–50 Ma. Preliminary results from stable oxygen and carbon isotope analyses, together with ^87^Sr/^86^Sr isotope data from calcite veins described above (Micheuz et al., 2018), indicate that vein calcite precipitated from seawater during late Eocene to Oligocene times. Accordingly, the related vein microstructures indicate that deformation is clearly postmagmatic, within the time range of the oldest recorded biostratigraphic age of the sediment cover at Sites U1439, U1440, U1441, and U1442 (late Eocene to earliest Oligocene, that is, ~35 Ma; Robertson et al., 2018). This implies a ~15 Ma hiatus between the formation of the igneous basement, forearc extension and the onset of (hemi) pelagic sedimentation (Robertson et al., 2018). The IBM forearc in general is sediment poor as it is widely remote from continental margin mass wasting sedimentation (e.g., Reagan et al., 2010; Robertson et al., 2018; Stern et al., 2003). Following Robertson et al. (2018), the hiatus was either controlled by topographic isolation, as indicated by epiclastic volcanic material at the basement‐cover contact at Site U1439, or sediments were bypassing the outer forearc to accumulate in the IBM trench. Fault‐controlled extensional basins, as observed at Expedition 352 drilling sites, therefore allowed the preservation of the sedimentary record with minimal reworking (Robertson et al., 2018) from ~35 Ma onward. The sedimentary and structural record at Sites U1439 and U1442 also suggests that displacement along confining normal faults was incremental, with a first episode of fault movement with syntectonic sedimentation during Time Slice 1 (as defined by Robertson et al., 2018) from circa 35 to 23 Ma.

**Figure 13:**
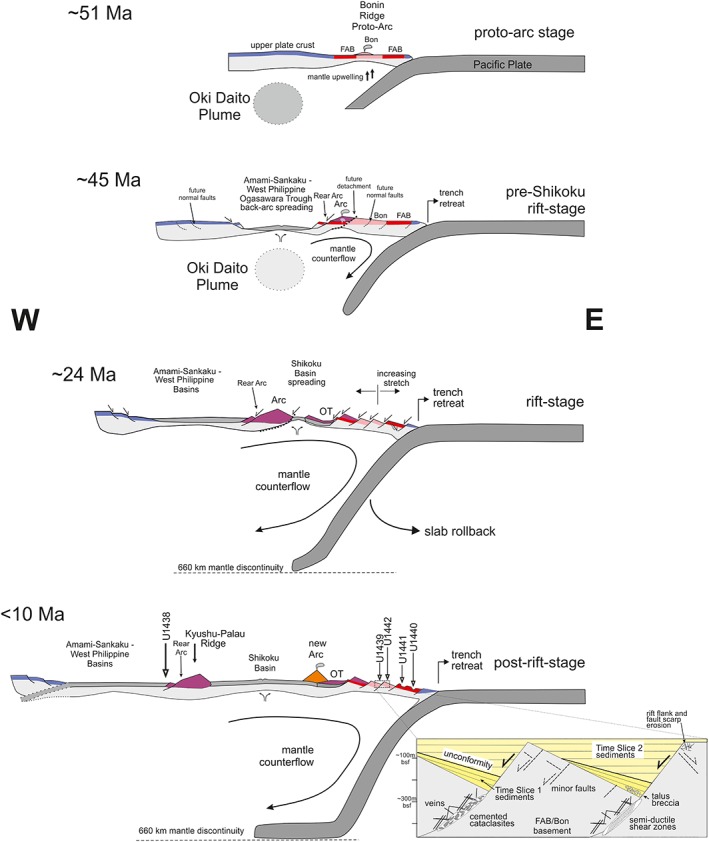
Schematic cross sections illustrating the evolution of the Philippine Sea plate near the latitude of IODP Expedition 352, based on Stern and Bloomer (1992), Ishizuka et al. (2006), Ishizuka, Tani, et al. (2011), Ishizuka, Taylor, et al., 2011), Wu et al. (2016), Brandl et al. (2017), Reagan et al. (2017), Faccenna et al. (2018), Ishizuka et al. (2018), Reagan et al. (2019), and this work. Not to scale. FAB (in red) = forearc basalt crust; Bon (in pink) = boninite crust; prearc crust is in blue; Arc = arc lavas with basaltic parents; OT = Ogasawara Trough. IODP Expedition 351 and 352 drill sites are shown on the <10 Ma panel. 51 Ma—approximately 1.5 Myr after subduction initiation and production of near‐trench FAB to Bon crust. 45Ma = backarc spreading at about the time that parental arc magmas transition from boninite to basalt. Initiation of spreading was at about 50 Ma while high‐Si boninite was erupting along the Ogasawara Ridge. At 24 Ma volcanism along the Kyushu‐Palau Ridge waned whereas the Shikoku Basin began to spread. Augmented subduction rollback and related trench retreat commenced when the Pacific slab reached the 660 km mantle discontinuity, from ~35 Ma onward. From <10 Ma steady state volcanism occurred along the present Izu‐Bonin volcanic arc. The inset shows the general tectonic and basin structure of the local forearc area, that is, normal faulting along cataclastic to semiductile shear zones, (half‐) grabens, and related synrift (Time Slice 1) and postrift (Time Slice 2) sedimentation, with an unconformity at ~27–23 Ma.

The boundary between Time Slices 1 and 2 coincides with the change of layering from subhorizontal above to inclined below 127 m bsf, respectively, at Site U1439. At Site U1442 ship‐board data (Reagan et al., 2015) show that bedding dip angles change from subhorizontal to up to 35° from 75 m bsf toward the basement contact. This angular discordance (Figure 13) can be correlated with an increase of dip angles at Site U1439 below 153 m bsf at ~27 Ma. The biostratigraphic data documented by Robertson et al. (2018) therefore provide a good constraint for this episode of faulting and related block tilting at circa 27–30 Ma (rift stage in Figure 13). This faulting increment immediately precedes the opening of the Shikoku‐Parece‐Vela basins at ~25 Ma (IIshizuka, Taylor, et al., 2011). It also coincides with a period of PSP regional extension that preceded focusing of extensional strain in the West Philippine backarc Basin, when spreading decreased and became more east‐west directed (Deschamps & Lallemand, 2002). For this interval the age‐depth plots for Site U1439 as described by Robertson et al. (2018) are characterized by a very steep to subvertical gradient indicating high sediment accumulation rates. Early to mid‐ Eocene sedimentation at Expedition 351 Site U1438, as documented from biostratigraphic data (Arculus, Ishizuka, Bogus, & the Expedition 351 Scientists, 2015; Barth et al., 2017) and U‐Pb detrital zircon ages, is not necessarily inconsistent with the evolution described above, as Site U1438 is located closer to the West Philippine Basin backarc spreading center, so that subsidence and sedimentation could have commenced earlier along the rear arc‐backarc transition zone.

At Sites U1439 and U1442 the greater part of the fault displacement therefore took place from circa 35 to 23 Ma, accompanied by synrift sedimentation (Figure 13). Tephras originally deposited horizontally show the same tilt as the associated sediment beds within Time Slice 1. The oldest tephra age within Time Slice 1 at Site U1439 is 32.3 Ma, whereas the youngest tephra age in Time Slice 1 is an ash dated at ~27 Ma (Kutterolf et al., 2018). Accordingly, a major increment of displacement and block tilting occurred at or immediately after 27 Ma. This was followed by waning displacement, with slow‐rift to postrift sediment deposition during the transition from rifting along the margins to spreading at the center of the Shikoku‐Parece‐Vela Basin system at 25 Ma (Ishizuka, Taylor, et al., 2011) (Figure 13). At that time spreading migrated from the West Philippine Basin, succeeded by the formation of the Shikoku and Parece‐Vela Basins (Deschamps & Lallemand, 2002; Faccenna et al., 2018) (Figures 1 and 13). These basins were subsequently formed by seafloor spreading during early and middle Miocene time, in a backarc position relative to the IBM subduction zone system (Kobayashi et al., 1995; Kutterolf et al., 2014) (Figure 13).

Our forearc basin analysis revealed a declining stretching gradient from the downslope sites (in particular Site U1441) toward the upslope sites (U1439 and U1442). Extension *e* is in the range of ~0.55 at Site U1441, with related strain rates of 3.31 × 10^−15^ to 9.66 × 10^−15^ s^−1^, in contrast to *e* ~ 0.175 with strain rates in the range of 1.58 × 10^−16^ to 4.62 × 10^−16^ s^−1^ at Sites U1439 and U1442. Normal faults that were located closer to the retreating trench (Figure 13) therefore underwent more stretching compared to the upslope extensional faults, which can be explained by flexure of the downslope forearc toward the retreating trench. In addition, the shearing at Sites U1440 and U1441 was accompanied by alteration, which resulted from fluid infiltration and related hydration reactions, potentially facilitating rheological weakening and an increase of strain rates during extension.

### 8.3 A Lithospheric Scale Tectonic Model Working Hypothesis

According to Reagan et al. (2019), subduction initiation at ~52 Ma was accompanied by rapid trench retreat and asthenospheric upwelling, followed by slab dehydration and melting of the depleted upper plate mantle. An early embryonic arc formed at ~51 Ma after termination of rapid trench retreat. Vast tectonic extension may therefore not be expected during a period of plate coupling, beginning about the time that the protoarc formed (i.e., ~51 Ma) (Figure 13). Discrete reverse faults, being later overprinted by younger extensional structures, are inferred to be related to that period of coupling between the subducting Pacific Plate and the IBM protoarc. Coupling presumably ended about the time of initial forearc extension and related basin formation ~15 Ma later.

With an assumptive average Pacific Plate subduction rate of 50 mm/year (e.g., Fryer, 1996; Fryer et al., 1990; Stern et al., 2003; Faccenna et al., 2009, 2018; Gong et al., 2018;Holt et al., 2018; Kong et al., 2018), and a subduction angle of ~30° along the northern IBM subduction segment (as indicated by seismic tomography, e.g., Jaxybulatov et al., 2013; Gong et al., 2018; Kong et al., 2018; Holt et al., 2018), the Pacific lower plate is expected to reach the 660 km mantle discontinuity within ~12–13 Ma after subduction initiation. Bending of the plate at this juncture can help to accommodate subduction rollback inception and decoupling along the plate boundary (Faccenna et al., 2009). Numerical models by Faccenna et al. (2009, 2018) and Čížková & Bina, 2013, Čížková & Bina, 2015) also show that subduction rollback commences within 10 to 20 Ma after subduction initiation. IBM forearc extension is therefore inferred to be related to Pacific slab rollback and the resulting trench retreat (Figure 13), presumably starting during Late Eocene times. In this case, the lack of sediment accumulation between ~50 and ~35 Ma would reflect the lack of accommodation space in the forearc, so that sediments bypassed into the trench.

Although the extensional strain rates in the range of 10^−16^ to 10^−15^ s^−1^ appear to be low, these rates just represent initial upper crust rifting of the IBM forearc. Toward the arc and the backarc (West Philippine spreading center; CBF rift of Ishizuka et al., 2018), rifting was assisted by magmatism probably related to the Oki Daito Plume (Ishizuka et al., 2018) (Figure 13), so that effective stresses dropped and the lithosphere was weakened locally (e.g., Koptev et al., 2017). Magmatism‐assisted rifting consequently resulted in a rheologically decoupled lithosphere, where initial brittle deformation was accumulated in the upper crust and subsequent lithospheric necking occurred due to weak lower crust and lithospheric mantle. The “boninite sites” (U1439 and U1442) are therefore situated between the more intensely stretched trench‐near downslope sites and the weakened arc/rear‐arc/backarc sites. Accordingly, these boninite sites form a less stretched, neutral domain between the retreating trench and the spreading arc and backarc. A major detachment potentially commenced along the upwelling mantle zone and the related plutons that were located beneath the IBM arc and the rear arc (Figure 13) as inferred by Stern (2010). This resulted in an increase in extensional strain that culminated in the rifting of the Ogasawara Trough during the Eocene time (Ishizuka et al., 2006) (Figure 13). Accordingly, spreading and arc magmatism ceased in the Amami‐Sankaku/West Philippine Basin and along the Kyushu‐Palau Ridge, respectively, as it was confined by this detachment, resulting in shutting down the arc (Stern, 2010). Subsequent spreading in the Shikoku Basin is likely to have left some of the original arc behind the Ogasawara Trough. The dismembered crust to the west of the Shikoku Basin, now exposed along the Kyushu‐Palau Ridge, therefore represents an arc‐derived allochthon that is now located along the western margin of the Shikoku‐Parece Vela Basin system. Continuous backarc spreading resulted in the formation of the Shikoku‐Parece Vela Basin from 25 Ma onward (IIshizuka, Taylor, et al., 2011).

### 8.4 Impacts on SSZ Ophiolite Analogues

The overall IBM forearc architecture as inferred from this study matches distinct structural features in ophiolites worldwide, especially those formed in a SSZ setting. SSZ ophiolites include the Tethyan ophiolites of the Alpine‐Mediterranean region (Beccaluva et al., 2004; Parlak, 2016; Robertson et al., 2002), the Cordilleran and western Pacific ophiolite complexes (Hopson et al., 2008; Robertson, 1989; Shervais et al., 2004; Snow & Shervais, 2015), and the Ordovician Appalachian‐Caledonian ophiolites (e.g., Bedard et al., 1998; Cawood & Suhr, 1992; Dewey & Casey, 2011; Jenner et al., 1991).

Most relevant here are SSZ ophiolites that preserve oceanic extensional structures and related sediments. The tuffaceous sedimentary cover of the Jurassic Coast Range ophiolite in central California (e.g., at Del Puerto Canyon) (Robertson, 1989; Hopson, & Mattinson, & Pessagno, 1981, Hopson, Mattinson, Pessagno, & Luyendyk, 2008) is comparable to the tuffaceous sedimentary cover of the Izu‐Bonin forearc (Kutterolf et al., 2018; Robertson et al., 2018). Unfortunately, the Coast Range ophiolite, like many other circum‐Pacific, Cordilleran‐type ophiolites, is too small in outcrop and overprinted by later active‐margin tectonics to easily recognize primary ocean‐floor extensional structures. Also, throughgoing ocean‐floor extensional structures are likely to have been reactivated during emplacement, so destroying any evidence of primary extensional tectonics. More promising are Tethyan ophiolites that are commonly preserved as huge intact sheets that may preserve ocean floor structures. Grabens and half‐graben formed parallel to the seafloor spreading center in the Late Cretaceous Troodos ophiolite (Varga & Moores, 1985; Allerton & Vine, 1990). Although not tuffaceous, metalliferous and pelagic sediments formed within ocean‐floor half‐grabens of similar scale and structure to those of the Izu‐Bonin forearc (Boyle & Robertson, 1984), in some parts several Ma after magmatic basement formation (e.g., Robertson, 1977). Similarly, in the Late Cretaceous Oman ophiolite, rifts orientated parallel (or obliquely) to the spreading center, notably the Alley structure (Smewing, 1980), hosted deep‐sea deposits including basaltic talus, metalliferous and pelagic sediments (Fleet & Robertson, 1980). In Oman, seafloor extensional (and transverse) structures were reactivated during ophiolite emplacement, associated with protrusion of serpentinized ultramafic rocks and subophiolite accretionary melange (Robertson & Woodcock, 1982). However, in contrast to the Izu‐Bonin forearc these extension‐related structures were short‐lived because subduction did not continue and create a related volcanic arc as associated tuffaceous sediments (e.g., Pearce & Robinson, 2010; Robertson et al., 2012).

Key features that SSZ ophiolites may share with the Izu‐Bonin forearc therefore include (1) basal metalliferous‐oxide sediments; (2) a hiatus between igneous basement formation and its deep‐sea sedimentary cover; (3) synmagmatic to postmagmatic extension, resulting in the formation of normal faults and related extensional structures, and fault‐bounded graben or half‐graben (Figure 13); and (4) a sedimentary cover that includes ophiolite‐derived talus and tuffaceous strata in small extensional basins.

## 9 Conclusions

The general structure of the fault‐bound sedimentary basins at IODP Expedition 352 drilling sites reflects the post Late Eocene tectonic evolution of the Philippine Sea Plate.

Extensional fault‐ and vein microstructures reveal the postmagmatic deformation conditions within the Izu‐Bonin forearc upper crust subsequent to the formation of its magmatic basement. Vein calcite microstructures indicate intracrystalline deformation within a low‐temperature‐high stress regime (*T* < ~250 °C; *σ* > 50 MPa). Mean differential stresses in the range of 70 to 90 MPa, calculated from calcite microstructures, were sufficient to exceed common tensile strength of the oceanic crust. Extension resulted in the formation of symmetric and asymmetric, fault‐bounded basins with initial syntectonic sedimentation. In the area of IODP Expedition 352 drilling sites, the IBM forearc was stretched by ~16–19% at the “boninite sites” and up to 55% at “FAB sites,” at strain rates in the range of 10^−16^ to 10^−15^ s^−1^. These rates are also indicated by the vein and fault zone microstructures.

The published magmatic ages from the IBM forearc basement, isotope data, and the biostratigraphic record from the cover sediments revealed a ~15 Ma hiatus between the rapidly forming near‐trench seafloor after subduction initiation around 52 Ma and subsequent tectonic forearc extension. We explain this 15 Ma time gap by ongoing rollback of the Pacific Plate that triggered upper plate extension. This was accommodated by normal faulting accompanied by extensional mineralized vein formation from late Eocene to early Oligocene times onward.

By introducing a superordinate general plate tectonic model, extension and arc spreading are inferred to have been tectonically controlled by Pacific Plate subduction rollback. Downslope increase of stretch is inferred to be related to upper plate flexure toward the retreating trench. Near the arc, rear arc, and the backarc, extension was potentially assisted by magmatism, resulting in advanced lithospheric extension around 27–26 Ma with subsequent spreading of the Shikoku and Parece Vela Basins (~25 Ma). This period coincides with a major unconformity within the sedimentary basin sequences. Parts of the IBM arc and rear arc, as well as the West Philippine Basin, were sheared off from the IBM forearc forming an allochthon that was delaminated from the IBM arc and rear arc. Along the IBM forearc this goes along with waning fault displacement and slow‐rift to postrift sediment deposition, and the transition from rifting along the margins to spreading at the center of the Shikoku‐Parece‐Vela basin system.

The overall IBM forearc architecture and development as inferred from this study may serve as a reference setting, in particular with regard to postmagmatic rift‐related structures and a sedimentary cover that includes tuffaceous strata and will help with the identification and interpretation of a SSZ origin of ophiolites worldwide.
